# Multivalent design of the monoclonal SynO2 antibody improves binding strength to soluble α-Synuclein aggregates

**DOI:** 10.1080/19420862.2023.2256668

**Published:** 2023-09-22

**Authors:** Inga Petersen, Muhammad Ilyas Ali, Alex Petrovic, Anders Jimmy Ytterberg, Karin Staxäng, Monika Hodik, Fadi Rofo, Sina Bondza, Greta Hultqvist

**Affiliations:** a Department of Pharmacy, Uppsala University, Uppsala, Sweden; b Department of Pharmacy, SciLifeLab Drug Discovery and Development, Uppsala University, Uppsala, Sweden; c TEM Laboratory, BioVis Platform, Uppsala University, Uppsala, Sweden; d Ridgeview Instruments AB, Uppsala, Sweden; e Department of Immunology, Genetics and Pathology, Uppsala University, Uppsala, Sweden

**Keywords:** Avidity, multivalent antibodies, Parkinson’s disease (PD), soluble aggregates, alpha-Synuclein (αsyn)

## Abstract

Soluble aggregates are reported to be the most neurotoxic species of α-Synuclein (αSyn) in Parkinson’s disease (PD) and hence are a promising target for diagnosis and treatment of PD. However, the predominantly intracellular location of αSyn limits its accessibility, especially for antibody-based molecules and prompts the need for exceptionally strong soluble αSyn aggregate binders to enhance their sensitivity and efficacy for targeting the extracellular αSyn pool. In this study, we have created the multivalent antibodies TetraSynO2 and HexaSynO2, derived from the αSyn oligomer-specific antibody SynO2, to increase avidity binding to soluble αSyn aggregate species through more binding sites in close proximity. The multivalency was achieved through recombinant fusion of single-chain variable fragments of SynO2 to the antibodies’ original N-termini. Our ELISA results indicated a 20-fold increased binding strength of the multivalent formats to αSyn aggregates, while binding to αSyn monomers and unspecific binding to amyloid β protofibrils remained low. Kinetic analysis using LigandTracer revealed that only 80% of SynO2 bound bivalently to soluble αSyn aggregates, whereas the proportion of TetraSynO2 and HexaSynO2 binding bi- or multivalently to soluble αSyn aggregates was increased to ~ 95% and 100%, respectively. The overall improved binding strength of TetraSynO2 and HexaSynO2 implies great potential for immunotherapeutic and diagnostic applications with targets of limited accessibility, like extracellular αSyn aggregates. The ability of the multivalent antibodies to bind a wider range of αSyn aggregate species, which are not targetable by conventional bivalent antibodies, thus could allow for an earlier and more effective intervention in the progression of PD.

## GRAPHICAL ABSTRACT

**Figure:**
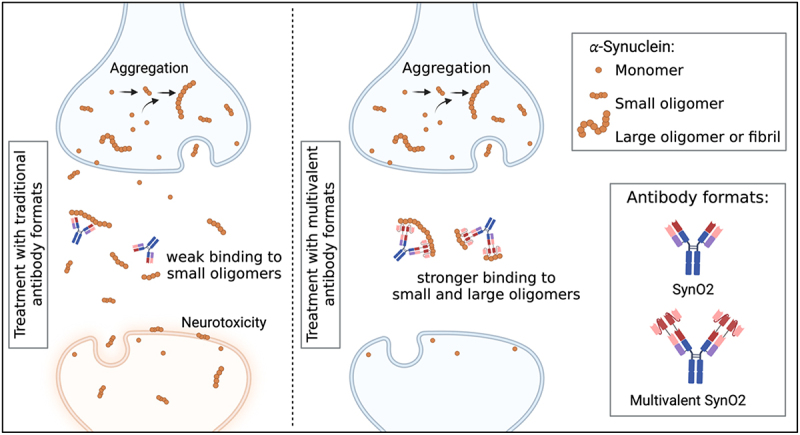

## Introduction

Aggregation and intracellular deposition of alpha-Synuclein (αSyn) are the main characteristics of neurodegenerative synucleinopathies, of which Parkinson’s disease (PD) is the most common. Whether αSyn aggregation is the disease’s cause or the result of another underlying misfunction is not fully understood, but the presence of αSyn aggregates in early presymptomatic PD stages and its contribution to the severe loss of dopaminergic neurons in the substantia nigra is widely accepted.^1^ The diagnosis of PD is still mostly carried out through the assessment of motor dysfunction.^2^ However, by the time PD becomes symptomatic approximately 30–50% of nigral dopaminergic neurons are already lost. Current medical treatments for PD patients compensate for the dopamine deficiency, but no disease-modifying treatment is available.

αSyn is located inside the pre-synaptic terminals of neurons as either disordered cytoplasmic monomers or helical membrane-bound monomers or multimers.^3–6^ Under physiological conditions, αSyn is presumably involved in the regulation of neurotransmitter release by acting as a chaperone on the SNARE (soluble N-ethylmaleimide-sensitive fusion protein attachment protein receptors) complex assembly.^4^ In PD pathology, αSyn aggregation is best described by the nucleation-conversion-polymerization model,^7,8^ where misfolded αSyn initiates nucleation, promoting a clustering of misfolded αSyn and resulting in oligomer formation. Evidence suggests that the oligomers have antiparallel β-sheet, i.e., hairpins,^9–11^ which twist to become parallel ordered β-sheet structures upon fibril formation.^7,12,13^ These fibrils exist in many different sizes and are predominantly insoluble, whereas oligomers are generally considered soluble.^8,14^

Many studies suggest that small αSyn oligomers are the most neurotoxic αSyn species, causing membrane perturbation,^15^ synaptic dysfunction,^16^ oxidative stress,^17,18^ mitochondrial dysfunction^19^ and neuronal inflammation.^20,21^ αSyn fibrils might also cause toxicity through the release of oligomers and seed further aggregation.^14^ It is likely that all αSyn species, monomers, oligomers and fibrils alike, are secreted by neurons to some degree and are partly found extracellularly.^22^ Extracellular αSyn oligomers and fibrils are reported to be taken up by phagocytic cells^23,24^ as well as by neurons, which has been suggested to contribute to the spread of αSyn pathology.^14,25,26^ Therefore, therapeutically targeting both αSyn oligomers and fibrils to reduce their toxicity and propagation has a high potential.

Utilizing antibodies as binders to αSyn aggregates offers high target specificity and affinity and has therefore been studied extensively for diagnostic and therapeutic applications in PD. Several antibodies targeting αSyn oligomers and fibrils have reached Phase 1 and Phase 2 clinical trials.^27–31^ These antibodies have been reported to reduce soluble plasma αSyn levels and slow down PD pathology in *in vitro* and *in vivo* models,^32^ but no therapeutic effect in humans has been observed in clinical trials thus far.^33^

High affinity binding of IgG antibodies to aggregates is in most cases achieved by avidity, i.e., the combined binding strength of multiple binding sites on one target molecule. Despite the small amount of αSyn extracellularly available for antibody binding, enhancing an antibody’s binding strength to soluble αSyn aggregates beyond bivalency could improve its sensitivity and efficacy for diagnostic and therapeutic applications by increasing the duration of antibody–target association. We have previously designed multivalent antibodies with the aim to increase the avidity to amyloid beta (Aβ) aggregates,^34,35^ where the hexavalent Hexa-RmAb158, derived from the antibody RmAb158 (murine version of lecanemab)^36^, showed an at least 40-fold stronger binding to Aβ protofibrils larger than 100 kDa compared to the bivalent RmAb158.^35^

In this study, we adopted the same multivalent antibody designs for the production of high-avidity αSyn aggregate-targeting antibodies, namely TetraSynO2 and HexaSynO2. Our designs are based on the antibody SynO2, which is reported to bind to αSyn oligomers and fibrils with a 27,000-fold higher affinity compared to monomers.^37^ Maintaining this low affinity to monomers is important to preserve the physiological function of monomeric αSyn^38^ and to minimize the risk of aSyn-antibody complexes being carried from the blood into the brain.

We show here that TetraSynO2 and HexaSynO2 exhibit increased avidity to soluble αSyn aggregates compared to SynO2, while retaining a low affinity to αSyn monomers, making the multivalent antibodies promising candidates for future diagnostic and treatment of PD.

## Results

### Generation of antibodies

**Figure 1.**
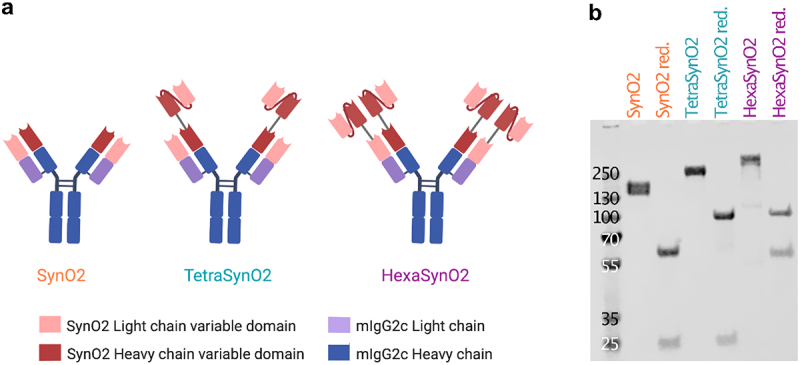
Design of recombinantly produced antibodies.

To increase avidity of SynO2 to αSyn oligomers and fibrils, single-chain variable fragments (scFv) of SynO2 were recombinantly fused to the N-terminal ends of each SynO2 heavy and light chain, forming HexaSynO2, or fused to only the N-terminal of the heavy chain, forming TetraSynO2 (Figure 1 a). The scFvSynO2 were recombinantly constructed from the SynO2 heavy and light chain variable domains linked together via a (G_4_S)_3_ linker. HexaSynO2, TetraSynO2 and the parental SynO2, used as a control, were expressed and purified yielding approximately 15 mg of SynO2, 2 mg of TetraSynO2 and 1 mg of HexaSynO2 per liter of transfected cell culture. SDS-PAGE analysis confirmed the size and purity of the purified antibodies, with SynO2 presenting as one band at 150 kDa, TetraSynO2 presenting as one band at ~ 200 kDa and HexaSynO2 presenting as one band at ~ 260 kDa (Figures 1b and S1). The heavy and light chains are clearly represented under reducing conditions, with the TetraSynO2 and HexaSynO2 having an expected elevated heavy chain molecular weight (MW) or heavy and light chain MW, respectively, compared to SynO2 (Figures 1 and S1).

(a) Schematic illustration of the parental antibody SynO2, TetraSynO2 and HexaSynO2 with scFvSynO2 recombinantly fused to the N-termini of heavy and/or light chains of SynO2. (b) SDS-PAGE with Coomassie staining of antibodies loaded under non-reducing and reducing (red.) conditions. Bands of intact antibodies under non-reducing conditions appear at approximately 150 kDa for SynO2, 200 kDa for TetraSynO2 and 260 kDa for HexaSynO2 (1 µg protein/lane).

### Recombinant fusion of scFvSynO2 on SynO2 minimally decreases thermal stability

**Figure 2.**
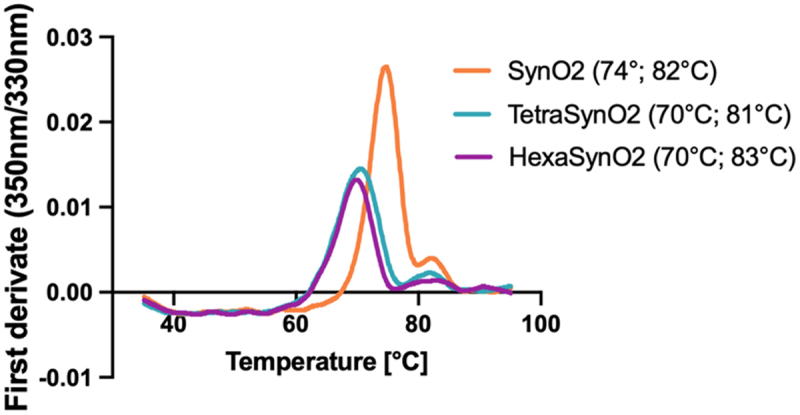
Thermal stability of SynO2, TetraSynO2 and HexaSynO2 measured by Tycho.

The addition of domains to an antibody can cause a change in its structural properties. We therefore tested the structural stability in increasing heat of SynO2, TetraSynO2 and HexaSynO2 using the Tycho nt.6 system. SynO2 showed high inflection temperatures of 74.4°C and 81.8°C (Figure 2), indicating a high structural stability. TetraSynO2 and HexaSynO2 gave very similar, but slightly lower first inflection temperatures of 70.2°C and 70.0°C, respectively (Figures 2 and S3). The lower inflection temperatures are likely due to the additional scFvs, which unfold at lower temperatures.^35,39^ However, the inflection temperatures are still relatively high, suggesting a high structural stability and an overall antibody-like folding of the tetra- and hexavalent antibody formats.

First derivate of ratio between intrinsic fluorescence measured at 350 nm and 330 nm, while the antibodies were heated up linearly from 35°C to 95°C. Inflection temperatures, visible as peaks, represent major unfolding events at 74°C and 82°C for SynO2, 70°C and 81°C for TetraSynO2 and 70°C and 83°C for HexaSynO2, suggesting high structural stability for all three antibodies.

### SynO2 selectivity for αSyn aggregates is based on avidity

**Figure 3.**
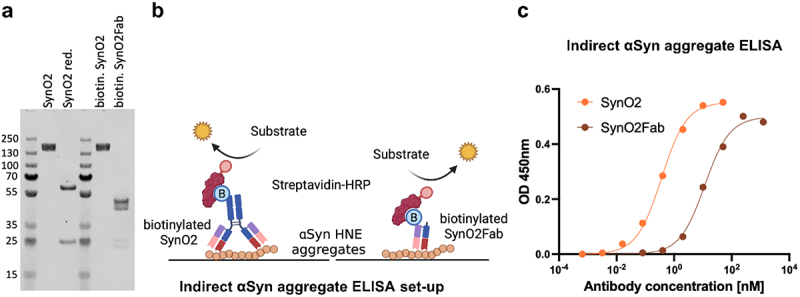
Characterization of SynO2Fab.

By creating TetraSynO2 and HexaSynO2 we aimed to increase the overall binding strength toward αSyn aggregates through enhanced avidity effects. We started out by determining whether the binding strength of SynO2 to aggregated αSyn is mediated by bivalent engagement of the antibody. To answer this question, we generated antigen-binding fragments (Fabs) of SynO2 (SynO2Fab) (Figure 3a) and compared their binding strength to αSyn aggregates, prepared by incubation with 4-hydroxy-2-nonenal (HNE), with the full SynO2 antibody in an indirect ELISA (Figure 3b, c). Coomassie-stained SDS-PAGE showed bands at the expected size for the freshly biotinylated SynO2 and SynO2Fab at 150 kDa and 50 kDa, respectively (Figure 3a). The indirect ELISA results showed approximately 100-fold weaker binding of SynO2Fab to αSyn HNE aggregates compared to the full antibody SynO2 (Figure 3c). These results suggest that avidity is crucial for the αSyn aggregate binding of SynO2.

(a) Coomassie-stained SDS-PAGE shows a band at 150 kDa for biotinylated SynO2 and a band at 50 kDa for the biotinylated SynO2Fab. 1 µg protein/lane. The complete gel can be seen in Figure S6. (b) Schematic illustration of the indirect αSyn aggregate ELISA set-up with biotinylated SynO2 or biotinylated SynO2Fab binding to the αSyn HNE aggregate coating and detection by Streptavidin-horseradish peroxidase (HRP). (c) Indirect αSyn aggregate ELISA binding curves show strong binding by SynO2 to αSyn HNE aggregates and weak binding by SynO2Fab. The degree of biotinylation of SynO2 and SynO2Fab was compared by direct ELISA (Figure S7) and was verified by liquid chromatography–mass spectrometry (Figure S8-9, Tables S1-3).

### HexaSynO2 binds amyloid beta aggregates weakly

**Figure 4.**
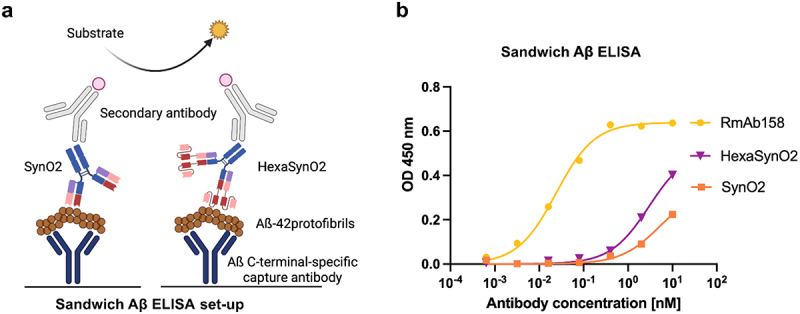
Sandwich Aβ ELISA detects cross-reactivity of SynO2 and HexaSynO2 with Aβ aggregates.

Aggregated αSyn shares properties with other amyloid proteins regarding their β-sheet secondary structure. To demonstrate that the high affinity of HexaSynO2 is not caused by an increased affinity toward a random structural element present in any amyloid aggregate, we tested its binding to Aβ aggregates present in Alzheimer’s disease using a sandwich ELISA with Aβ42 protofibrils captured by an Aβ42 C-terminal-specific antibody (Figure 4a). The results showed that both SynO2 and HexaSynO2 bind to Aβ, but only when applied at elevated concentrations (Figure 4b). HexaSynO2 had slightly stronger binding to Aβ, compared to SynO2, but still demonstrated a 100-fold weaker binding when compared to the Aβ aggregate-specific antibody RmAb158. Despite having weak binding to Aβ aggregates, these results suggest an unspecific mode of binding of SynO2 and HexaSynO2 to Aβ aggregates.

(a) Schematic illustration of the sandwich Aβ ELISA set-up with Aβ42 protofibrils captured by an Aβ C-terminal-specific antibody coated to a plate. Binding of SynO2, HexaSynO2 and RmAb158 was detected through an HRP-conjugated secondary antibody. (b) Sandwich ELISA binding signal demonstrating unspecific binding of SynO2 and HexaSynO2 to Aβ42 protofibrils at high antibody concentrations. Nonlinear regression curves (“one site – specific binding”) were calculated in GraphPad Prism.

### HexaSynO2 has a higher apparent affinity to αSyn HNE aggregates than SynO2

**Figure 5.**
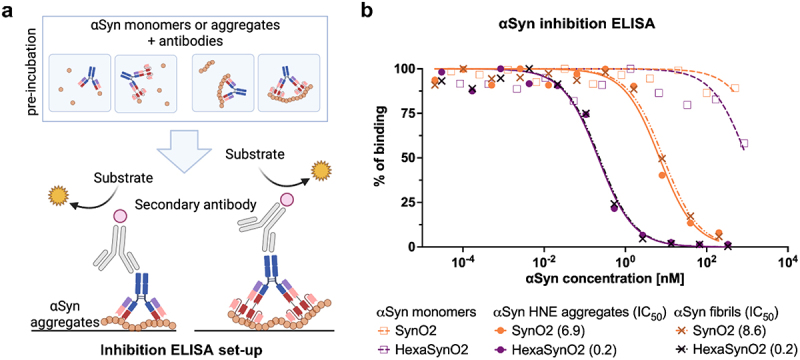
Inhibition ELISA illustrating the binding strength of SynO2 and HexaSynO2 to αSyn monomers, HNE aggregates and αSyn fibrils.

As HexaSynO2 features more αSyn binding sites than SynO2, we expected it to have increased binding strength to αSyn HNE aggregates and αSyn fibrils due to increased avidity compared to SynO2. An inhibition ELISA setup (Figure 5a) was chosen to detect differences in the binding strength of these antibodies. In contrast to classic indirect ELISA setups, the inhibition ELISA has the advantage of incubating the antibodies with αSyn in solution, which resembles a more physiologically relevant situation. Additionally, binding signals in classic ELISAs are measured after long incubation times of the antibody with its target to achieve a binding equilibrium and are therefore not sensitive enough to detect significant differences in binding strength between very strong binders.

(a) Schematic illustration of the αSyn inhibition ELISA set-up, with SynO2 or HexaSynO2 pre-incubated with αSyn monomers, αSyn HNE aggregates or αSyn fibrils, and subsequently added to an αSyn HNE aggregate-coated plate. (b) Visualization of binding signals, normalized to 0% as no signal and 100% as the maximal signal of each construct, respectively. Linear regression curves (“log(inhibitor) vs. normalized response”) were calculated in GraphPad Prism. IC50 values, calculated from the regression curves, indicate the concentration of αSyn HNE aggregate or αSyn fibrils needed in solution with the antibody to inhibit 50% of the respective antibody from binding to the αSyn HNE aggregate-coated surface.

With the inhibition ELISA we observed a 34-fold and 43-fold stronger binding of HexaSynO2 to αSyn HNE aggregates and αSyn fibrils, respectively, when compared to SynO2. Both, SynO2 and HexaSynO2 exhibited very low binding to αSyn monomers (Figure 5b). The binding strength was measured as the half-maximal inhibitory concentrations (IC_50_), which is defined as the concentration of target proteins in the pre-incubation mixture at which 50% of the antibodies are inhibited from binding to the target immobilized on the plate (Figure 5b).

### TetraSynO2 and HexaSynO2 dissociate slower from αSyn HNE aggregates than SynO2

**Figure 6.**
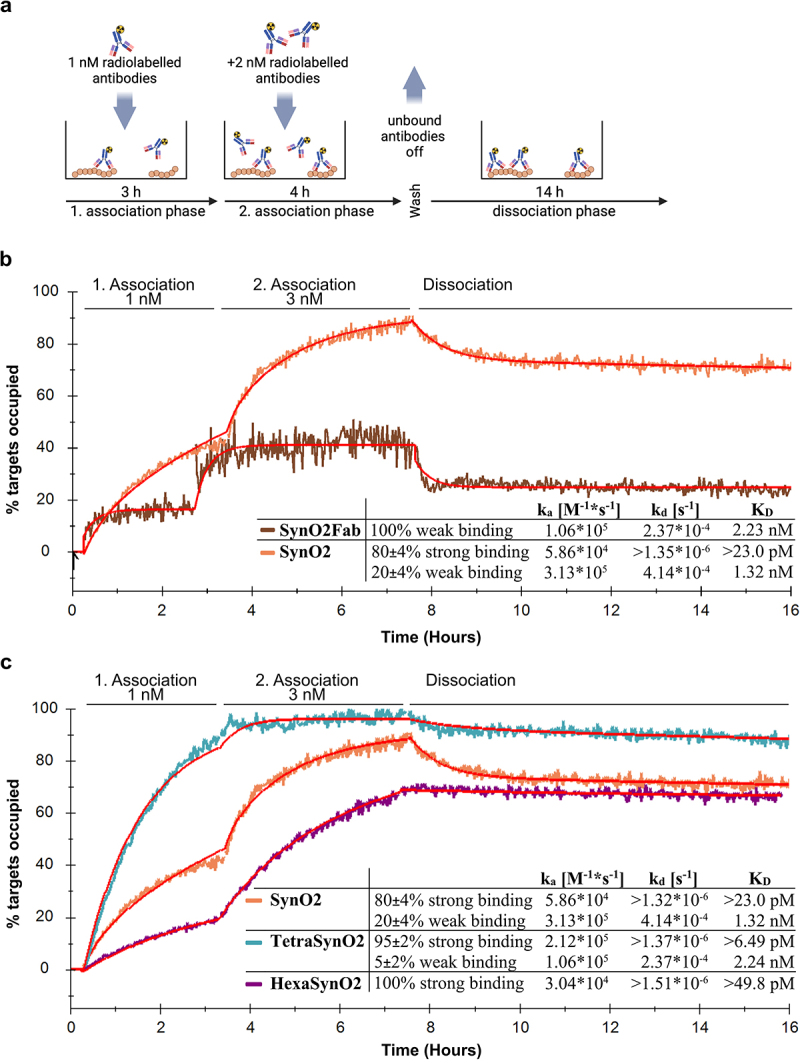
Kinetic evaluation of interactions of SynO2Fab, SynO2, TetraSynO2 and HexaSynO2 with αSyn HNE aggregates recorded by LigandTracer.

LigandTracer assays were conducted to test whether the increased affinity of the multivalent antibody format to αSyn HNE aggregates, as seen in the inhibition ELISA, leads to a decreased dissociation rate as expected if it has achieved increased avidity. Compared to other techniques measuring protein binding kinetics, such as surface plasmon resonance, LigandTracer allows the determination of the kinetics of very strong interactions, as protein dissociations can be measured over an extended period of time. Iodine-125 (^125^I)-labeled antibodies were applied to αSyn HNE aggregate-coated dishes at two different concentrations consecutively to achieve more accurate calculations of the kinetic parameters. The dissociation kinetics were subsequently measured after all unbound antibodies were removed (Figure 6a).

(a) Schematic illustration of the LigandTracer set-up with a plastic surface coated with αSyn HNE aggregates and ^125^I-labeled antibodies added in solution. (b) Interaction curves of SynO2Fab and SynO2, and (c) SynO2, TetraSynO2 and HexaSynO2 with αSyn HNE aggregates recorded by LigandTracer and fitting curves (red) using a one-to-one depletion corrected model (SynO2Fab, HexaSynO2) or a one-to-two model (SynO2, TetraSynO2), respectively. 100 nM coating with αSyn HNE aggregates. Two consecutive association phases (3 hours and 4 hours, respectively) with 1 nM and 3 nM of the respective ^125^I-labeled antibody. Recorded interaction curves were evaluated in TraceDrawer using a “one-to-one depletion corrected” (SynO2Fab and HexaSynO2) or a “one-to-two” model (SynO2 and TetraSynO2). Signal intensities of each curve were scaled to Bmax, the estimated signal intensity at saturation, with 100% representing target saturation.

Different kinetic models were used to evaluate the kinetic parameters of each interaction. The interaction of SynO2Fab with αSyn HNE aggregates was well described by a “one-to-one depletion corrected” interaction model. Correction for ligand depletion was justified by the theoretical ratio of αSyn HNE aggregates to SynO2Fab being 5:1 at the highest ligand concentration, which was also visible in the rapid curve saturation in both association phases (Figure 6b). The dissociation rate constant (k_d_) calculated for SynO2Fab using the “one-to-one depletion corrected” model was 2.37 × 10^−4^ s^−1^, reflecting the k_d_ of a monovalent interaction (Figure 6b).

For the interaction of the full SynO2 antibody with αSyn HNE aggregates, we observed a biphasic dissociation for which a “one-to-one” interaction model was not sufficient. The biphasic dissociation indicates a heterogeneous interaction with overlapping weak (monovalent) and strong (bivalent) components, which has been shown to be best described by using a “one-to-two” interaction model.^40^ Here, the k_d_ calculated for the weaker of the two interaction components of SynO2 was 4.14 × 10^−4^ s^−1^, which coincides with the affinity calculated for the monovalent SynO2Fab, indicating that this weak interaction component of SynO2 is a monovalently binding cohort of antibody molecules. The k_d_ of the stronger interaction component of SynO2 was calculated to be at least 1.35 × 10^−6^ s^−1^, which is a 300-fold slower k_d_ than the weak interaction component (Figures 6b and S10a). The strong interaction component likely represents a cohort of SynO2 that binds bivalently to the target, contributing approximately 80 ± 4% to the overall binding.

The interaction of TetraSynO2 with αSyn HNE aggregates also showed a partially biphasic dissociation, though with a smaller cohort of quickly dissociating antibodies. Using a “one-to-two” model with the kinetic parameters for the weaker interaction component set constant to the association rate constant (k_a_) and k_d_ values obtained with SynO2Fab resulted in a k_d_ of at least 1.37 × 10^−6^ s^−1^ for the stronger interaction component, similar to the k_d_ obtained for the bivalently binding SynO2 cohort (Figures 6c and S10b). Similar kinetic values were obtained for TetraSynO2 using a “one-to-one” model, indicating that the cohort of TetraSynO2 antibodies binding monovalently represents only a minor fraction. The contribution of avidity enhanced binding to the overall binding of TetraSynO2 was estimated to be 95 ± 2%.

HexaSynO2 showed a homogeneous, stable interaction with αSyn HNE aggregates with nearly no dissociation, indicating that all antibodies bound bi- or multivalently to the target. We used a “one-to-one depletion corrected” model for the kinetic evaluation, which resulted in similar kinetic parameters as a “one-to-one” model, indicating that ligand depletion is only present to a minor extent, but described the interaction curve of the first association phase more accurately. The calculated k_d_ of at least 1.51 × 10^−6^ s^−1^ was again similar to the values seen with the strong interaction components of TetraSynO2 and SynO2 (Figure 6c).

However, we cannot exclude that the true dissociation rates of the strong interaction components, presented above, are even slower. Since the slowest dissociation rates that we could measure here resulted in a horizontal dissociation line, dissociation rates slower than that could not be distinguished. Even if the kinetic evaluation were run with the dissociation rates locked to 10^−8^ s^−1^, we achieved equally good fitting curves. Hence, the dissociation rates for the strong interaction components presented here should be considered at least in the range of 10^−6^ s^−1^, but could be stronger.

In conclusion, the LigandTracer results support our hypothesis presented above (Figure 3c) that SynO2 binds αSyn HNE aggregates strongly, due to its ability to bind them with higher avidity. The increased avidity in the multivalent antibody formats decreases their dissociation from the target even further and increases the cohort size of antibodies being able to bind bi- or multivalently to αSyn HNE aggregates.

## Discussion

Here, we developed the tetravalent antibody, TetraSynO2, and the hexavalent antibody, HexaSynO2, with the aim to increase the avidity of SynO2 to αSyn aggregates, such as oligomers and fibrils. We thereby intended to target a higher amount of soluble αSyn aggregates using the same antibody dose. Previously, a similar multivalent antibody design was successfully used with the Aβ aggregate-specific antibody RmAb158.^34,35^

SynO2, the antibody that we based the multivalent antibody designs upon, was originally reported to bind conformation-specific αSyn oligomers and that the αSyn C-terminal was part of the antibody’s epitope.^37^ By ELISA and LigandTracer assays, we showed that the monovalent SynO2Fab had a much lower affinity to αSyn HNE aggregates compared to the full antibody. Furthermore, our inhibition ELISA results showed that SynO2 had almost identical affinities to αSyn HNE aggregates and αSyn fibrils, which both appeared structurally very different in transmission electron microscopy analysis (Fig. S4e). Thus, our findings suggest that the specificity of SynO2 toward αSyn aggregates is dependent upon avidity binding, rather than depending solely on a structural epitope.^41^ Increasing the number of binding sites and having them in close proximity, as in TetraSynO2 and HexaSynO2, should further increase the antibodies’ avidity to αSyn aggregates, assuming that the aggregates are large enough to accommodate bivalent binding of the antibodies to the same aggregate at the same time.

For a detailed characterization of the binding kinetics, we carried out LigandTracer experiments for which the αSyn HNE aggregates had to be immobilized to the dish surface. The coating concentration was aimed at the lower range of 100 nM (1.4 µg/ml), but still high enough to ensure good signal/noise ratio. Under these experimental conditions, we could show that the proportion of antibodies binding bi- or multivalently to αSyn HNE aggregates increased from 80 ± 4% for the parental antibody SynO2 to 95 ± 2% for TetraSynO2 and estimated close to 100% for HexaSynO2, based upon the contribution of the strong interaction component to the overall interaction (Figure 6c). We defined the strong interaction components for TetraSynO2 and HexaSynO2 by their slow dissociation rate, which were calculated to be at least 170- or 300-fold higher, respectively, compared to the dissociation rate calculated for the weak interaction components. Hence, the additional scFvSynO2 in TetraSynO2 and HexaSynO2 allowed proportionally more antibodies to bind bi- or multivalently to αSyn HNE aggregates. With the antibody Hexa-RmAb158, we have previously shown that the additional binding sites and their close proximity in the hexavalent format not only increased the antibody’s binding strength to large Aβ aggregates compared to the bivalent RmAb158, but also enabled strong binding to small Aβ oligomers, to which RmAb158 had only weak affinity.^35^ Similar to Hexa-RmAb158, we also expect HexaSynO2 to offer more flexibility to the antibody, allowing it to bind conformations and sizes of αSyn aggregates that would sterically not be accessible to SynO2.

We could not accurately measure any interaction stronger than bivalent binding with our experimental set-up, as the slowest dissociation rate for bivalent interactions was calculated to be in the range of 10^−6^ s^−1^ and already resulted in a horizontal line during dissociation. All dissociation rates calculated here for the strong interaction components should therefore be considered at least as slow as stated, if not slower. Hence, it was not possible for us to draw any further conclusion about the valency of the interaction other than distinguishing between monovalent and any higher valency binding. In contrast to the substantial changes observed for the dissociation rates, the association rates varied only to a minor degree between the different antibody formats and interaction components, implying similar molecular target recognition, and therefore indicating that the same epitope is recognized.

For cases where multivalent binding establishes quickly relative to the measurement time, the 1:2 model presents a suitable approach to distinguish the antibody fractions that engage in monovalent versus multivalent binding. The 1:2 model assumes the presence of two independent 1:1 interactions, which is not entirely accurate for discriminating between the monovalent and avidity enhanced binding.^40^ For multivalent binders the number of available target epitopes decreases faster than predicted for a 1:1 interaction, which is reflected in a seemingly slower on-rate. This is one of the reasons why the strong binding components, which represent bi- or multivalent interactions, have lower k_a_-values compared to the weaker, monovalent interactions. Another factor contributing to the small differences in association rates is that the binder is either a scFv of SynO2 or an original variable domain without additional linkers. Adding an amino acid linker between the variable heavy and light chain of an antibody to generate an scFv can result in structural changes and has been reported previously to alter its affinity compared to the antibody’s original binding site.^42^ Therefore, the use of scFvSynO2 as binding sites in TetraSynO2 and HexaSynO2 could explain the slight decrease in molecular recognition observed for the multivalent constructs. For HexaSynO2, the initial binding event is more likely to occur through a scFvSynO2, since it has relatively more scFvs than original binding sites. It is also likely that the two original αSyn binding sites on HexaSynO2 are partly sterically hindered by the attached scFvs, and therefore are less accessible to participate in αSyn aggregate binding, which might also explain the lower k_a_-value observed for the multivalent binding fraction. In TetraSynO2, however, the original binding sites are likely to be more accessible, as the scFv are only attached to the heavy chain, which may be the reason why TetraSynO2 exhibits a slightly higher k_a_-value for the multivalent fraction compared to HexaSynO2.

In conclusion, the limited amount of αSyn aggregates present extracellularly, along with the wide spectrum of αSyn aggregates sizes and structures, poses a challenge in targeting the propagation of toxic αSyn aggregates in the brain. By avidity-enhanced binding, traditional bivalent antibody formats can discriminate well between physiologically important monomeric and pathological aggregated targets, but may fail to bind with avidity to all different aggregate species. Here we have shown that by introducing additional binding sites to the antibody SynO2, the proportion of antibodies binding by avidity to αSyn aggregates was improved to approximately 100%, resulting in even stronger binding. This study underlines the importance of a detailed evaluation and understanding of the antibody binding kinetics when dealing with a limited and diverse target pool. With their enhanced binding strength to large αSyn moieties, the multivalent antibodies TetraSynO2 and HexaSynO may have an improved diagnostic and therapeutic potential against αSyn aggregation, spreading, and toxicity.

## Material and methods

### Antibody cloning, expression and purification

scFv of the SynO2 antibody were designed with an internal (G_4_S)_3_ linker and recombinantly fused with a 20 amino acid linker (RADAAPGGGSGGGTVSIFPP) to the N-terminus of the variable region of both heavy and light chain of the parental SynO2 antibody to generate a hexavalent antibody, HexaSynO2. The genes for heavy and light chain were cloned into pcDNA3.4 vectors (GeneArt, Regensburg, Germany). Antibodies were expressed by transient transfection in Expi293 cells as described previously^43^. In brief, vectors for light and heavy chain were transfected at a ratio of 7:3 with polyethyleneimine (PEI) (Polysciences 24,765–1) as a transfection agent. Seven days post-transfection, cell culture supernatants were filtered through a 0.22 µm filter (Millipore GPW04700) and antibodies were purified from the supernatant by affinity chromatography using a protein G column (Cytiva GE17-0404-01). Antibodies eluted at approx. 70% elution buffer (0.7% acetic acid). Purified antibodies were concentrated using a 30K MWCO Amicon Ultra-15 centrifugal filter unit (Millipore UFC9030) and the buffer was changed to phosphate-buffered saline (PBS) using a 7K MWCO Zeba spin desalting column (Thermo Scientific 89,892). Protein purity was validated by analytical size exclusion chromatography (SEC), for which 10–50 µl of antibodies in PBS (at the respective concentrations of 0.7 mg/ml for SynO2; 0.26 mg/ml for TetraSynO2 and 0.2 mg/ml for HexaSynO2) were loaded at 0.5 ml/min on a Superdex 200 Increase 10/300 GL, equilibrated with PBS, using an Äkta Go system (Cytiva, Uppsala, Sweden).

### Protein stability analysis by Tycho

The protein stability was analyzed using a Tycho nt.6 instrument (NanoTemper Technologies, Munich, Germany), where proteins were loaded into glass capillaries and heated up to 95°C, while their intrinsic fluorescence at 330 and 350 nm was measured. Structural changes cause changes in the amount of tyrosine and tryptophan exposed, which result in a shift in the fluorescent intensity. Major unfolding events are measured as inflection temperatures, indicated as peaks in the first derivate of the fluorescence intensity ratio 350/330 nm.

### Fab preparation

Fabs were generated using the Pierce Fab Micro preparation Kit (Thermo Scientific 44,685) following the manufacturer’s instructions. In brief, the immobilized Papain slurry was washed and activated with Fab digestion buffer containing 20 mM cysteine pH7. The buffer of the antibodies was changed to 20 mM cysteine Fab digestion buffer using a Zeba spin desalting column 7K MWCO (Thermo Scientific 89,882). The antibodies (~0.8 µg/µl concentration) were incubated with the immobilized papain, shaking at 37°C for 15 min. The digests were separated from the immobilized papain and purified on a Nab Protein A Plus Spin Column (Thermo Scientific 89,952). The flowthroughs containing the Fabs were concentrated using a 10K MWCO Amicon Ultra-0.5 Centrifugal concentrator (Millipore UFC5003), and the buffer was exchanged to PBS using a 7K MWCO Zeba spin desalting column (Thermo Scientific 89,882). The Fab was further purified by SEC, for which the Fab in PBS was loaded at 0.5 ml/min on a Superdex 200 Increase 10/300 GL, and elution fractions corresponding to 50 kDa MW were pooled and concentrated.

### Generation of αSyn HNE aggregates and αSyn fibrils and separation by size exclusion chromatography

αSyn aggregates were prepared by incubation with HNE as described previously.^44^ Briefly, 1 mg lyophilized αSyn monomers (AnaSpec AS-55555) were dissolved to 13 mM (2 mg/ml) in 50 mM phosphate buffer pH 7.4 and centrifuged for 5 min at 21,000×g at 4°C to remove preformed insoluble aggregates. 465 µl of αSyn monomers at 13 mM were mixed with 35 µl of 64 mM HNE (10 mg/ml) (Cayman chemicals 32,100), vortexed for 10 s and incubated at 37°C for 72 hours without agitation. The solution was again centrifuged for 5 minutes at 21,000×g at 4°C, and the buffer was changed to PBS using a Zeba spin desalting column 7K MWCO (Thermo Scientific 89,882). αSyn fibrils were prepared by incubation of αSyn monomers (AnaSpec AS-55555) (13 mM in 50 mM phosphate buffer pH 7.4) at 37°C shaking for 7 days. The αSyn fibrils were centrifuged for 10 min at 21,000 × g at 4°C, and pellets, containing the insoluble fibrils, were resuspended in PBS. Single-use aliquots of αSyn HNE aggregates and αSyn fibrils were stored at −80°C. SEC was performed with αSyn HNE aggregates to separate large and small αSyn aggregates into individual fractions. αSyn HNE aggregates were thawed on ice, insoluble aggregates were removed by centrifugation for 5 min at 21,000×g at 4°C and 500 µl protein solution were loaded on a Superdex 200 Increase 10/300 GL at a flow rate of 0.6 ml/min using the Äkta pure 25 system (Cytiva, Uppsala, Sweden). Fractions of 500 µl were collected and analyzed by SDS-PAGE, Native PAGE and western blot.

### Transmission electron microscopy of αSyn HNE aggregates and αSyn fibrils

A 5 µl drop of αSyn HNE aggregates, ~1.7 mg/ml diluted 1:2 in MQ water, or αSyn fibrils, ~1.7 mg/ml undiluted, was placed on a formvar- and carbon coated 200-mesh copper grid (Ted Pella). The excess solution was removed by blotting with filter paper. The sample was then directly contrasted with 2% uranyl acetate. Excess of uranyl acetate was removed by blotting on filter paper. Images were acquired on a Tecnai™ G2 Spirit BioTwin transmission electron microscope (Thermo Fisher/FEI) at 80 kV with an ORIUS SC200 CCD camera and Gatan Digital Micrograph software (both from Gatan Inc./Blue Scientific).

### SDS-PAGE and western blot analysis

The purity and size of purified proteins were determined by SDS-PAGE analysis. 1 µg purified protein was loaded with 1× LDS sample buffer (Invitrogen B0007) with or without 1× Bolt Sample reducing agent (Invitrogen B0009) on a Bolt 4 to 12% Bis-Tris 1 mm protein gel (Invitrogen NW04125) alongside a pre-stained protein marker (Thermo Scientific 26,619). The proteins were separated by size at 80 V for 1–2 hours. PAGE blue protein solution (Thermo Scientific 24,620) was used to stain for total protein. The identity of protein bands was confirmed by western blot. The proteins were transferred from the unstained SDS-PAGE gel to a PVDF membrane (Thermo Scientific 88,518) for 2 hours at 100 V using 1× Transfer buffer (Invitrogen BT00061) containing 20% methanol. The membrane was dried, reactivated in methanol and blocked with 5% skim milk and 1% Tween in Tris-buffered saline (TBS). If αSyn HNE aggregates or αSyn fibrils were blotted, an additional step to fixate aggregates on the membrane was added after reactivating the membrane, including a 30-min incubation of the membrane in 0.5% PFA in PBS. The membrane was washed in PBS, TBS-Tween, and blocked subsequently in 5% skim milk and 1% Tween in TBS. Primary and secondary antibodies chosen for the respective proteins, indicated in the respective result sections, were incubated with the membrane for 1 h. Either fluorescent signal or chemiluminescent signal from the reaction of an HRP-conjugated secondary antibody with ECL substrate (Invitrogen WP20005) was recorded with the Odyssey Fc Imaging system (LI-COR Biosciences, Lincoln, NE). Signal intensity analysis was carried out using ImageStudio Lite Software (LI-COR Biosciences, Lincoln, NE).

### Native PAGE analysis

Native PAGE was performed to analyze the protein size under non-denaturing conditions. 1–2 µg purified protein was loaded with 1× Native PAGE sample buffer (Invitrogen BN2003) on a NativePAGE 4 to 16% Bis-Tris 1 mm protein gel (Invitrogen BN1002) alongside an unstained protein standard (Invitrogen LC0725). 1× Native PAGE running buffer as anode buffer and 1× Native PAGE running buffer with 1:200 diluted Native PAGE cathode additive as cathode buffer were used. The proteins were separated at 80 V for 2 hours. Native PAGE gels were destained and fixed in 40% methanol with 10% acetic acid. PAGE blue protein solution (Thermo Scientific 24,620) was used to stain for total protein. Western blot with Native PAGE was done as described above with SDS-PAGE.

### Biotinylation of antibodies

Antibodies or Fabs were biotinylated using EZ-Link^TM^ Sulfo-NHS-Biotin (Thermo Scientific A39257) according to the manufacturers instructions. The antibody or Fab at concentrations of 0.7 mg/ml and 0.5 mg/ml, respectively, was incubated with a 50-fold molar excess of biotin for 30 min at room temperature (RT). Unbound biotin was removed using a 7K MWCO Zeba spin desalting column (Thermo Scientific 89,882) equilibrated with PBS.

### Labelling of antibodies with iodine-125

Antibodies were radioactively labeled with iodine-125 (^125^I) according to the Chloramine T method.^45^ 20 µg of each antibody were mixed with 4 MBq ^125^I (PerkinElmer Inc., Waltham, MA) and 5 µl of 1 mg/ml Chloramine-T (Sigma-Aldrich 857,319) and incubated for 90 seconds. 10 µl of 1 mg/ml sodium metabisulfite (Supelco 08982) was added to stop the reaction. The labeled antibodies were purified from free ^125^I using a 7K MWCO Zeba spin desalting column (Thermo Scientific 89,882) equilibrated with PBS.

### Indirect ELISA to analyze avidity of SynO2 to soluble αSyn aggregates

The binding ability of SynO2 and SynO2Fab toward αSyn HNE aggregates was determined by indirect ELISA with biotinylated SynO2 and SynO2Fab. A high binding half-area 96-well plate (Corning CLS3690) was coated with 10 nM of αSyn HNE aggregates (molar concentration of monomeric units) in PBS overnight at 4°C. The following day, the coating solution was removed and the plates were blocked with 1% bovine serum albumin (BSA; Sigma-Aldrich A7030) in PBS for 2 hours shaking at RT. Serial dilutions of the antibodies were prepared in ELISA incubation buffer (0.1% BSA, 0.05% Tween-20 in PBS), with a starting concentration of 10 nM for SynO2 or 1000 nM for SynO2Fab, and incubated on the plate for 2-hour shaking at RT. HRP-conjugated Streptavidin (MAbTech 3310-9-1000) was added at a concentration of 1:4,000 and incubated for 1-hour shaking at RT. The HRP-substrate K-blue aqueous TMB (Neogen 331,177) was incubated on the plate for 1 min and the reaction was stopped by the addition of 1 M sulfuric acid. The signal intensity was measured as absorbance at 450 nm on the FLUOstar Omega microplate reader (BMG Labtech, Ortenberg, Germany) or on the TECAN Spark plate reader (Tecan, Männedorf, Switzerland). All antibody dilutions were prepared in ELISA incubation buffer (0.1% BSA, 0.05% Tween-20 in PBS), and wells were washed between each incubation with ELISA washing buffer (0.05% Tween-20 in PBS).

### Sandwich ELISA to assess cross-reactivity of HexaSynO2 and SynO2 toward Aβ42 aggregates

To investigate whether the additional binding sites on HexaSynO2 increase the reactivity to amyloids other than αSyn, we tested the binding of HexaSynO2 and SynO2 toward Aβ42 aggregates in a Sandwich ELISA. A high binding half-area 96-well plate (Corning CLS3690) was coated with a C-terminal Aβ42-specific antibody (Invitrogen 700,254) at a concentration of 1 µg/ml in PBS overnight at 4°C. After blocking the plates for 2 hours with 1% BSA (Sigma-Aldrich A7030) in PBS, cross-linked Aβ42 aggregates were added at a concentration of 10 nM in ELISA incubation buffer (0.1% BSA, 0.05% Tween-20 in PBS) and incubated for 2 hours shaking at RT. Serial dilutions of the antibodies in ELISA incubation buffer, with a starting concentration of 10 nM, were incubated on the plates for 2-hour shaking at RT. Antibody binding was detected and read as described above.

### Inhibition ELISA to discriminate the binding between αSyn monomers, αSyn HNE aggregates and αSyn fibrils

Inhibition ELISA was performed to evaluate the differences in binding strength of HexaSynO2, TetraSynO2 and SynO2 to αSyn monomers, soluble aggregates and fibrils. A high binding half-area 96-well plate (Corning CLS3690) was coated with αSyn HNE aggregates at a concentration of 100 nM in PBS at 4°C over night and was blocked the next day with 1% BSA (Sigma-Aldrich A7030) in PBS for 2 hours shaking at RT. Serial dilutions of αSyn monomers, αSyn HNE aggregates and αSyn fibrils, starting with 2000 nM of monomers or 200 nM of HNE aggregates and αSyn fibrils, were pre-incubated with 500 pM of HexaSynO2, TetraSynO2 or SynO2 in low-binding plates for 1.5 hours shaking at RT. The pre-incubated antibody-αSyn samples were added to the αSyn aggregates-coated plate and incubated for 15 min shaking at RT. Antibodies that were tightly bound to the αSyn species in solution were washed off subsequently, while unbound antibodies or those with weaker binding to the αSyn species in solution would be susceptible for binding to the αSyn aggregates coating increasingly with decreasing concentration of αSyn in the pre-incubation mixture. HRP-conjugated goat-anti-mouse antibody was added to detect antibodies bound to the plate and signals were developed and read as described above. IC50 of αSyn in solution, at which the antibody-binding to the coating was reduced by 50%, was calculated in GraphPad, where αSyn concentrations were log transformed and the OD_450_ values were normalized with OD_450_ values of zero set as 0% binding and the highest OD_450_ value, respectively, set as 100% binding. Linear regression curves were calculated using the “log(inhibitor) vs. normalized response” model.

### Real-time interaction analysis with LigandTracer

Association and dissociation rates of the antibodies to their target were determined using LigandTracer gray (Ridgeview Instruments, Uppsala, Sweden), which determines the amount of ^125^I-labeled ligand binding to a surface-bound target by measuring the radioactivity in a defined “target area”, coated with the target, and a background area, located opposite to each other on a Petri dish. In the LigandTracer instrument, the Petri dish is placed on an inclined, rotating platform. Due to the incline, only the lower part of the Petri dish stays covered with the buffer. During a run, the platform rotates 180° every 30 seconds, alternating the incubation of the target area and the background with the buffer. The radioactivity is recorded in the upper part of the Petri dish which is not covered by buffer. 300 µl of 100 nM αSyn HNE aggregates in PBS were used to coat the target area on a Petri dish (Sarstedt 83.3902) overnight at 4°C. The next day, the coating solution was removed and the Petri dish was blocked with 5% BSA (Sigma-Aldrich A7030) in PBS for 2 hours at RT. The blocking solution was replaced by 2 ml 0.1% BSA in PBS. The Petri dish was placed in the LigandTracer and the background signal was measured for 10 min. The buffer was replaced by 2 ml 0.1% BSA where 1 nM ^125^I-labeled antibody was added and the first association phase was run for 3 hours. A second association phase was performed with 3 nM ^125^I labeled antibody for 4 hours followed by a dissociation phase overnight, where 2 ml 0.1% BSA in PBS without antibody was incubated on the plate. Overlay images, fitting curves and kinetic parameters were calculated with the TraceDrawer software (Ridgeview Instruments, Uppsala, Sweden). For the kinetic evaluation, start values for the respective kinetic parameters were set as summarized in Table 1. For easy visual comparison, signal intensities were scaled based on the estimated signal at saturation, Bmax (100/Bmax). The percentage of antibodies binding by avidity in the overall binding was calculated by Bmax1/(Bmax1+Bmax2), with Bmax1 representing the signal at saturation of the avidity enhanced interaction component and Bmax2 representing the signal at saturation of the weaker interaction component.

**Table 1.** Start values for kinetic evaluations of interactions between antibodies and αSyn HNE aggregates recorded by LigandTracer. All interaction curves were individually fit with start values set at ^(1)^ global scope or ^(2)^ constant scope as indicated below, respectively.

	k_a_1 (M^−1^* s^−1^)	k_d_1 (s^−1^)	k_a_2 (M^−1^* s^−1^)	k_d_2 (s^−1^)	nB	Vol (L)
SynO2Fab	1.0e5^(1)^	1.0e-3^(1)^	-	-	2e12^(1)^	2.0e-3^(2)^
SynO2	1.0e5^(1)^	3.0e-4^(1)^	3.0e5^(1)^	1.0e-3^(1)^	-	-
TetraSynO2	1.0e5^(1)^	3.0e-6^(1)^	1.06e5^(2)^	2.37e-4^(2)^	-	-
HexaSynO2	1.0e5^(1)^	1.0e-3^(1)^	-	-	2e12^(1)^	2.0e-3^(2)^

## Supplementary Material

Supplemental Material

Supplemental Material

## Abbreviations

^125^I: Iodine-125
Aβ: amyloid beta
αSyn: alpha-Synuclein
Bmax: maximal binding capacity
BSA: bovine serum albumin
ELISA: enzyme-linked immuno assay
Fab: antigen-binding fragments
HMW: high molecular weight
HNE: 4-hydroxynonenal
HRP: horseradish peroxidase
IC50: Half-maximal inhibitory concentrations
k_a_: association rate constant
k_d_: dissociation rate constant
KD: affinity
kDa: kilo dalton
MW: molecular weight
MWCO: molecular weight cutoff
PBS: phosphate-buffered saline
PD: Parkinson’s disease
PEI: polyethyleneimine
red.: reducing
RT: room temperature
scFv: single-chain variable fragments
SDS-PAGE: Sodium dodecyl-sulfate polyacrylamide gel electrophoresis
SEC: size exclusion chromatography
SNARE: soluble N-ethylmaleimide-sensitive fusion protein attachment protein receptors
TBS: Tris-buffered saline
TEM: transmission electron microscopy
