# Supplementary material for: Multivalent design of the monoclonal SynO2 antibody improves binding strength to soluble α-Synuclein aggregates
Source: MAbs. 2023 Sep 22;15(1):2256668. doi: 10.1080/19420862.2023.2256668 (PMC10519360; doi:10.1080/19420862.2023.2256668)
Supplement: Supplemental Material [file KMAB_A_2256668_SM1409.docx]

**Figure captions and Alt texts – Supplementary**

**Supplementary Figure S1.** **Complete image of Coomassie-stained SDS-PAGE presented in Figure 1 b.** Antibodies were loaded under non-reducing and reducing (red.) conditions. Bands of intact antibodies under non-reducing conditions appear at approximately 150 kDa for SynO2, 200 kDa for TetraSynO2 and 260 kDa for HexaSynO2. 1 µg protein/lane. Lanes marked with * are irrelevant for the project.

**Supplementary Figure S1 Alt Text.** Complete image of Coomassie-stained SDS-PAGE gel presented in Figure 1 b.

**Supplementary Figure S2.** **Size exclusion chromatography (SEC) with (a) SynO2, (b) TetraSynO2 and (c) HexaSynO2.** Injected protein amounts were 50 µg for SynO2, 28 µg for TetraSynO2 and 10 µg for HexaSynO2 (respective concentrations were: SynO2 0,7 mg/ml; TetraSynO2 0,26 mg/ml; HexaSynO2 0,2 mg/ml). The protein absorbance was measured at 280 nm.

**Supplementary Figure S2 Alt Text.** (A-C) Three size exclusion chromatogramms with the antibodies SynO2, TetraSynO2 and HexaSynO2. The absorbance at 280 nanometer wavelength is plotted against the retention volume. All three antibodies eluted in one major peak, respectively, indicating a purity of 90 to 100 percent with no significant amount of aggregation.

**Supplementary Figure S3.** **Thermal stability of SynO2, TetraSynO2 and HexaSynO2 measured by Tycho.** Raw data of ratio between intrinsic fluorescence measured at 350 nm and 330 nm, while protein was heated up linearly from 35°C to 95°C.

**Supplementary Figure S3 Alt Text.** Raw data of Tycho measurements of the antibodies SynO2, TetraSynO2 and HexaSynO2. The ratio between intrinsic fluorescence measured at 350 nanometer and 330 nanometer wavelengths is plotted against the temperature. The first derivate of these data are shown in Figure 2.

**Supplementary Figure S4.** **Characterization of αSyn HNE aggregates and αSyn fibril preparations.** (a) SDS-PAGE with Coomassie staining with a band at ~15 kDa in the lane with αSyn monomers, bands at ~15 kDa and >250 kDa in the lane with HNE aggregates, and bands at ~15 kDa, ~ 40 kDa and >250 kDa in the lane with fibrils. 1 µg protein/lane. (b) Western blot of SDS PAGE with a band at ~15 kDa in the lane with αSyn monomers, bands at ~15 kDa, ~ 40 kDa and >250 kDa in the lane with αSyn HNE aggregates, and a ladder of bands between 15 kDa and >250 kDa in the lane with the αSyn fibril preparation. 0,25 µg protein/lane. Detection with Syn1 as primary antibody which binds all types of αSyn species. (c) Western blot of native PAGE of αSyn HNE with a band in the high molecular weight (HMW) range. 0,5 µg protein/lane. Syn1 was used as detection antibody. The complete gels and blots can be seen in Supplementary Fig. S5. (d) SEC elution chromatogram of αSyn HNE aggregates in PBS separated on a Superdex 200 Increase 10/300 GL column. The elution peaks measured by the absorbance at 280 nm indicate the presence of HMW species (calculated MW >700 kDa) and monomers (calculated MW 90 kDa). (e) TEM with negative staining shows αSyn HNE aggregates as elongated curly structures and fibrils as straight elongated structures.

**Supplementary Figure S4 Alt Text.** A-C show SDS-PAGE and western blots of SDS-PAGE and Native PAGE with alpha-Synuclein monomers, HNE aggregates, and fibrils. **Figure S4a.** SDS-PAGE with Coomassie staining of alpha-Synuclein monomers, HNE aggregates, and fibrils. Monomers appear as a single band at approximately 15 kilo Dalton while the majority of HNE aggregates and fibrils run in the high molecular weight range (above 250 kilo Dalton). **Figure S4b.** Western blot of alpha-Synuclein monomers, HNE aggregates, and fibrils separated by SDS-PAGE. Monomers appear as a single band at approximately 15 kilo Dalton. HNE aggregates and fibrils also have a weak band at approximately 15 kilo Dalto, but the majority of HNE aggregates appears in the high molecular weight range, while the fibrils ladder of bands between 15 kilo Dalton and above 250 kilo Dalton. **Figure S4c.** Western blot of alpha-Synuclein monomers, HNE aggregates, and fibrils separated by Native PAGE. The monomers appear as a single band at approximately 15 kilo Dalton, while the HNE aggregates appear as a single band in the higher molecular weight range. **Figure S4d.** Size exclusion chromatogram of alpha-Synuclein HNE aggregates. The absorbance at 280 nanometer wavelength is plotted over the retention volume. The major elution peak indicates that the majority of HNE aggregates have a molecular weight larger than 700 kilo Dalton. **Figure S4e.** Transmission electron microscopy images with negative staining of alpha-Synuclein HNE aggregates and alpha-Synuclein fibrils. Both HNE aggregates and fibrils appear elongated but the HNE aggregates are curly whereas the fibrils straight.

**Supplementary Figure S5. Complete images of PAGEs and blots presented in Supplementary Figure S4 a-c.** (a) SDS-PAGE with Coomassie staining with αSyn monomers, αSyn HNE aggregates and αSyn fibrils. 1 µg protein/lane. Lanes marked with * are irrelevant for the project. (b) Western blot of SDS-PAGE with αSyn monomers, αSyn HNE aggregates and αSyn fibril preparation. 0,25 µg protein/lane. Detection with Syn1 as primary antibody which binds all types of αSyn species. (c) Western blot of native PAGE of αSyn monomers and αSyn HNE aggregates. 0,5 µg protein/lane. Syn1 was used as detection antibody.

**Supplementary Figure S5 Alt Text.** a-c. Complete images of Coomassie-stained SDS-PAGE gel and western blots presented in Figure S4 a-c.

**Supplementary Figure S6. Complete image of Coomassie-stained SDS-PAGE presented in Figure 3 a.** Bands at 150 kDa for SynO2 and at 50 kDa for the Fab fragment SynO2Fab. A band at 25 kDa likely represents separated light and heavy Fab chains, likely caused by the reduction by cysteines in the Fab digestion buffer. 1 µg protein/lane. Lanes marked with * are irrelevant for the project.

**Supplementary Figure S6 Alt Text.** Complete image of Coomassie-stained SDS-PAGE gel presented in Figure 3 a.

**Supplementary Figure S7. Direct ELISA comparing the degree of biotinylation of SynO2 and SynO2Fab.** A dilution series of biotinylated SynO2 or SynO2Fab was applied as coating and the biotinylation was detected by Streptavidin-HRP. The Fab fragment appears to have a slightly lower degree of biotinylation but the difference is far below the difference between the binding signals of biotinylated SynO2 and SynO2Fab to αSyn aggregates shown in Figure 3.

**Supplementary Figure S7 Alt Text.** The readout of a direct ELISA with biotinylated SynO2 and biotinylated SynO2Fab is presented in a line graph. The absorbance at 450 nanometer wavelength is plotted over the antibody concentration. The two binding curves differ only to a small degree, suggesting that the degree of biotinylation of both antibodies is comparable.

**Supplementary Figure S8. Determination of the neutral, average mass using LC-MS.** Reduced, biotinylated Fab was measured by LC-MS. The left figure (A) shows the LC trace (total ion chromatogram, with two major peaks). The spectra of the left peak (indicated by a red arrow), containing the light chain, were combined into the spectrum in (B). The spectrum contains four major charge state envelopes, formed by varying number of ionizing protons from the ionization, which can be deconvoluted into neutral average masses in (C).

**Supplementary Figure S8 Alt Text.** The procedure for determining the intact mass of proteins. **Figure S8A.** LC-chromatogram with the light chain from reduced, biotinylated SynO2Fab. **Figure S8B.** Mass spectrum with the charge state envelope. **Figure S8C.** The deconvoluted spectrum.

**Supplementary Figure S9. Determination of the neutral, average mass using LC-MS.** The figure shows the deconvoluted masses of reduced SynO2 (A-B), reduced SynO2Fab (C-D) and intact SynO2Fab (E) measured by LC-MS. The intensities are based on the charge states envelopes in the raw spectra. The intact masses of all protein forms with matched masses are indicated in the figure and listed in Supplementary Tables 1-3. Red arrows indicate peaks shifted by the mass of a biotin moiety (339.5 Da). The intact, reduced heavy chain from SynO2 (B), contains N-linked glycosylation, where the G0F (red) and G1F (blue) constitute the major forms. The schematic carbohydrate structures are shown in the upper left inset in figure (B), with the key to the right. The intensities of the deconvoluted protein forms were used to calculate the distribution of biotin moieties per SynO2 and SynO2Fab, and compared to the measured number of biotin moieties (F). Blue corresponds to calculated values for SynO2, red for calculated SynO2Fab, and grey for measured SynO2Fab. The y-axis shows the relative amount in percent and the x-axis the number of biotin moieties. The data was used to calculate the average number of biotin moieties per SynO2 and SynO2Fab (F, inset).

**Supplementary Figure S9 Alt Text.** LC-MS spectra used to determine the average number of biotinylations of intact SynO2 and SynO2Fab. Peaks shifted by the mass of a biotin moiety are indicated. **Figure S9A.** Deconvoluted spectrum of the light chain from reduced, biotinylated SynO2. **Figure S9B.** Deconvoluted spectrum of the heavy chain from reduced, biotinylated SynO2. **Figure S9C.** Deconvoluted spectrum of the light chain from reduced, biotinylated SynO2Fab. **Figure S9D.** Deconvoluted spectrum of the heavy chain from reduced, biotinylated SynO2Fab. **Figure S9E.** Deconvoluted spectrum of the intact, biotinylated SynO2Fab. **Figure S9F.** A bar chart showing the distribution of biotinylations of calculated and/or measured SynO2 and SynO2Fab with the relative amount plotted against the number of biotins. The distribution has its peak at approximately 3 biotins, at which the relative amount of molecules is approximately 25 percent. The calculated average number of biotinylations is presented with 3.2 biotins for SynO2 and 2.8 biotins for SynO2Fab.

**Supplementary Figure S10.** Interaction curves of (a) SynO2 and (b) TetraSynO2 with αSyn HNE aggregates recorded by LigandTracer with one-to-two fitting curves split into the weak (light red) and the strong (dark red) interaction component, respectively. 100 nM coating with αSyn HNE aggregates. Two consecutive association phases (3 hours and 4 hours respectively) with 1 nM and 3 nM of the respective 125I-labelled antibody. Recorded interaction curves were evaluated in TraceDrawer using a. Signal intensities of each curve were scaled to Bmax, the estimated signal intensity at saturation, with 100% representing target saturation.

**Supplementary Figure S10 Alt Text.** Interaction curves of SynO2 and TetraSynO2 with alpha-Synuclein HNE aggregate recorded by LigandTracer are plotted as shown in Figure 6 but with additionally the one-to-two fitting curve split into its two components, which represent the weak and the strong interaction component.
