# Supplementary material for: Multivalent design of the monoclonal SynO2 antibody improves binding strength to soluble α-Synuclein aggregates
Source: MAbs. 2023 Sep 22;15(1):2256668. doi: 10.1080/19420862.2023.2256668 (PMC10519360; doi:10.1080/19420862.2023.2256668)
Supplement: Supplemental Material [file KMAB_A_2256668_SM1404.docx]

**Supplementary material for:**

**Multivalent design of the monoclonal SynO2 antibody improves binding strength to soluble 𝛼-Synuclein aggregates**

Inga Petersen^1^, Muhammad Ilyas Ali^1^, Alex Petrovic^1^, Anders Jimmy Ytterberg^2^, Karin Staxäng^3^, Monika Hodik^3^, Fadi Rofo^1^, Sina Bondza^4,5^, Greta Hultqvist^1^

*^1^Department of Pharmacy, Uppsala University, Sweden*

*^2^Department of Pharmacy, SciLifeLab Drug Discovery and Development, Uppsala University, Sweden*

*^3^TEM Laboratory, BioVis Platform, Uppsala University, Sweden*

*^4^Ridgeview Instruments AB, Vänge, Sweden*

*^5^Department of Immunology, Genetics and Pathology, Uppsala University, Sweden*

Corresponding author: Greta Hultqvist. Email: greta hultqvist@farmaci.uu.se

**
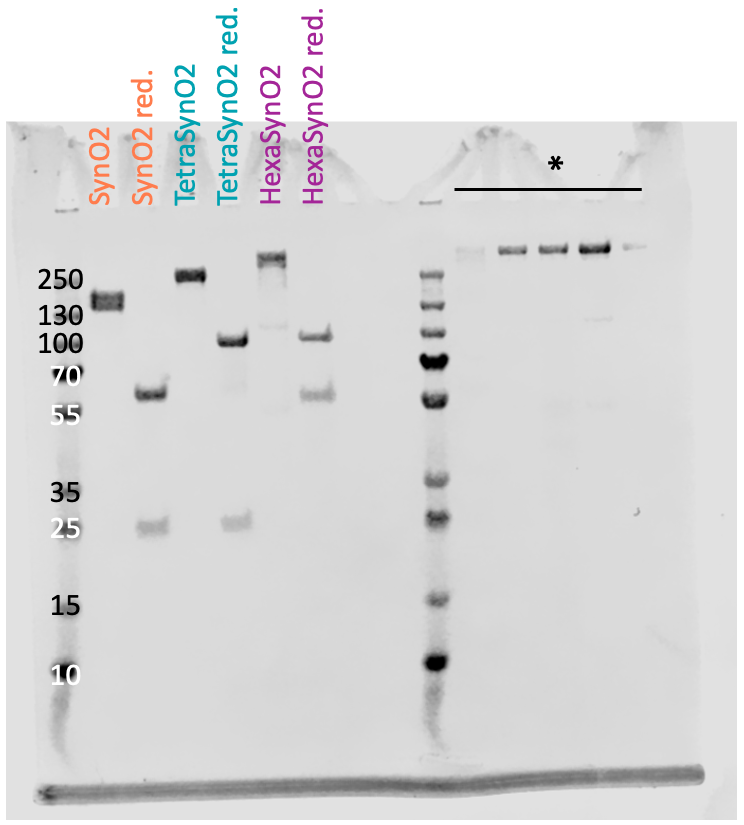
**

**Supplementary Figure S1. Complete image of Coomassie-stained SDS-PAGE presented in Figure 1 b.** Antibodies were loaded under non-reducing and reducing (red.) conditions**.** Bands of intact antibodies under non-reducing conditions appear at approximately 150 kDa for SynO2, 200 kDa for TetraSynO2 and 260 kDa for HexaSynO2. 1 µg protein/lane. Lanes marked with * are irrelevant for the project.

**a**

**
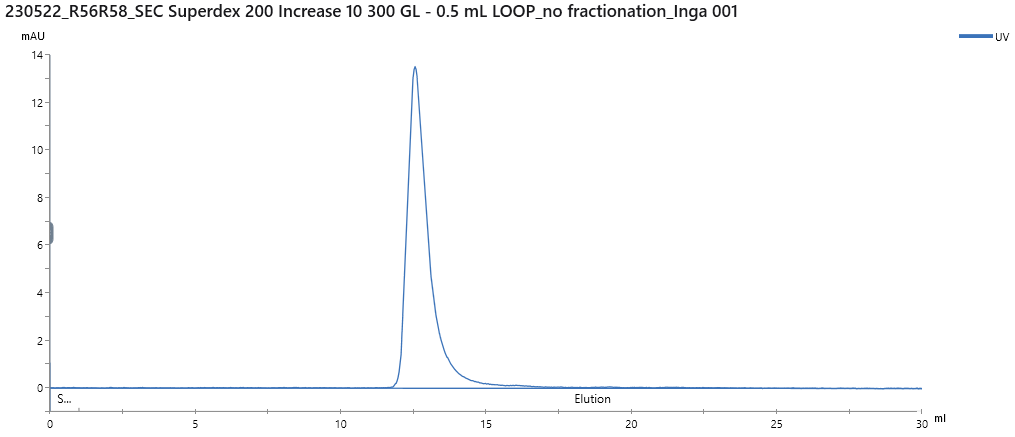
**

**b**

**
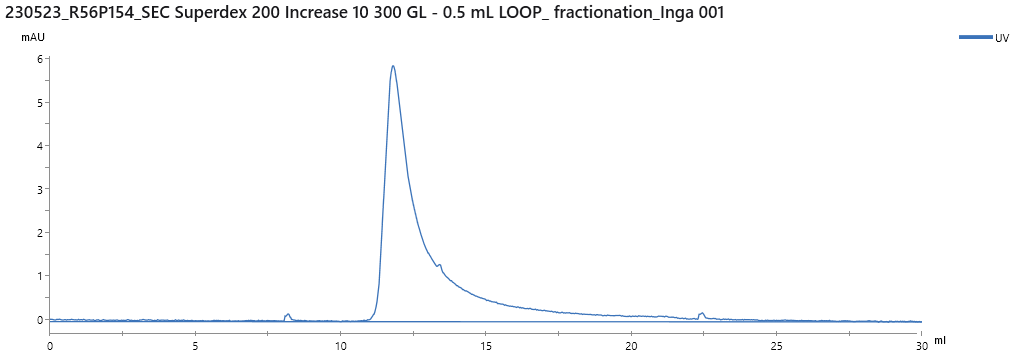
**

**c
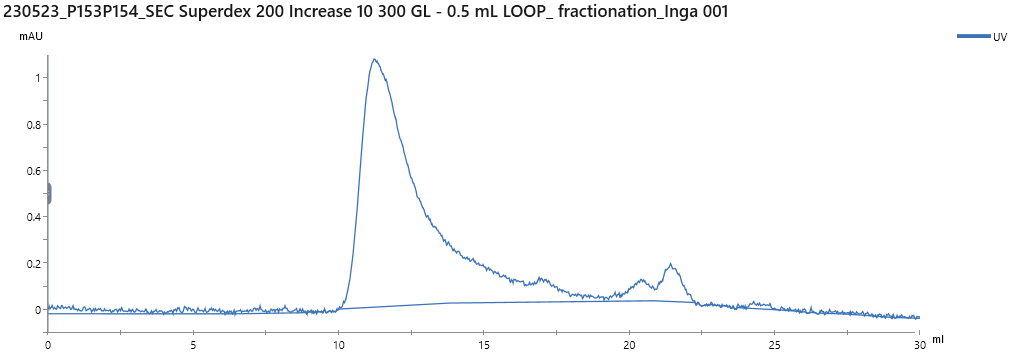
**

**Supplementary Figure S2. Size exclusion chromatography (SEC) with (a) SynO2, (b) TetraSynO2 and (c) HexaSynO2.** Injected protein amounts were 50 µg for SynO2, 28 µg for TetraSynO2 and 10 µg for HexaSynO2 (respective concentrations were: SynO2 0,7 mg/ml; TetraSynO2 0,26 mg/ml; HexaSynO2 0,2 mg/ml). The protein absorbance was measured at 280 nm.


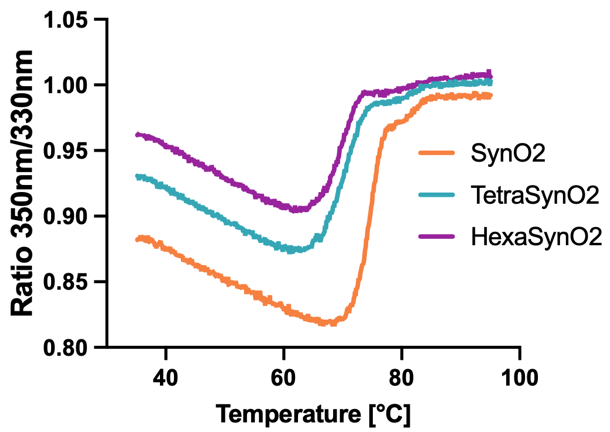


**Supplementary Figure S3. Thermal stability of SynO2, TetraSynO2 and HexaSynO2 measured by Tycho.** Raw data of ratio between intrinsic fluorescence measured at 350 nm and 330 nm, while protein was heated up linearly from 35°C to 95°C.

***αSyn HNE aggregates have a distinctly different structure from insoluble fibrils***

To test the antibodies’ binding kinetics *in vitro* to αSyn aggregates, we generated two types of αSyn aggregates *in vitro*. 1. αSyn monomers were incubated with the cross-linker 4-hydroxynonenal (HNE), which has previously been reported to produce a range of soluble αSyn aggregates at various sizes.^1^ 2. Non-soluble fibrils were generated from αSyn monomers by shaking without cross-linking agent.

An SDS-PAGE run with freshly dissolved monomeric αSyn revealed a strong band of monomers at the expected size of 15 kDa (Suppl. Fig. S4 a). For both the αSyn HNE aggregates and the αSyn fibril preparations, the majority of the proteins appeared to be at the very top of the Coomassie-stained SDS-PAGE gel (Suppl. Fig. S4 a), suggesting the presence of large aggregated species. Western blot analysis with Syn1 or SynO2 as detection antibodies, revealed an additional monomeric band, along with αSyn oligomeric species at various sizes, ranging from dimers up to high molecular weight (HMW) species in the αSyn HNE aggregates and the αSyn fibril preparations (Suppl. Fig. S4 b). Further analysis of the αSyn HNE aggregates by Native PAGE western blotting (Suppl. Fig. S4 c) and size exclusion chromatography (SEC) (Suppl. Fig. S4 d) confirmed the previous observation that the HNE aggregates were predominantly HMW aggregates of sizes larger than 700 kDa, with only a small proportion consisting of monomers. Structural differences between the differently prepared aggregates were observed using transmission electron microscopy (TEM), where the fibril preparation contained long, straight structures, while the HNE aggregate preparation contained curly, elongated structures of different shapes (Suppl. Fig. S4 e). Our findings here indicate that the αSyn aggregate preparation with HNE produced large soluble αSyn aggregates of elongated shapes.

**
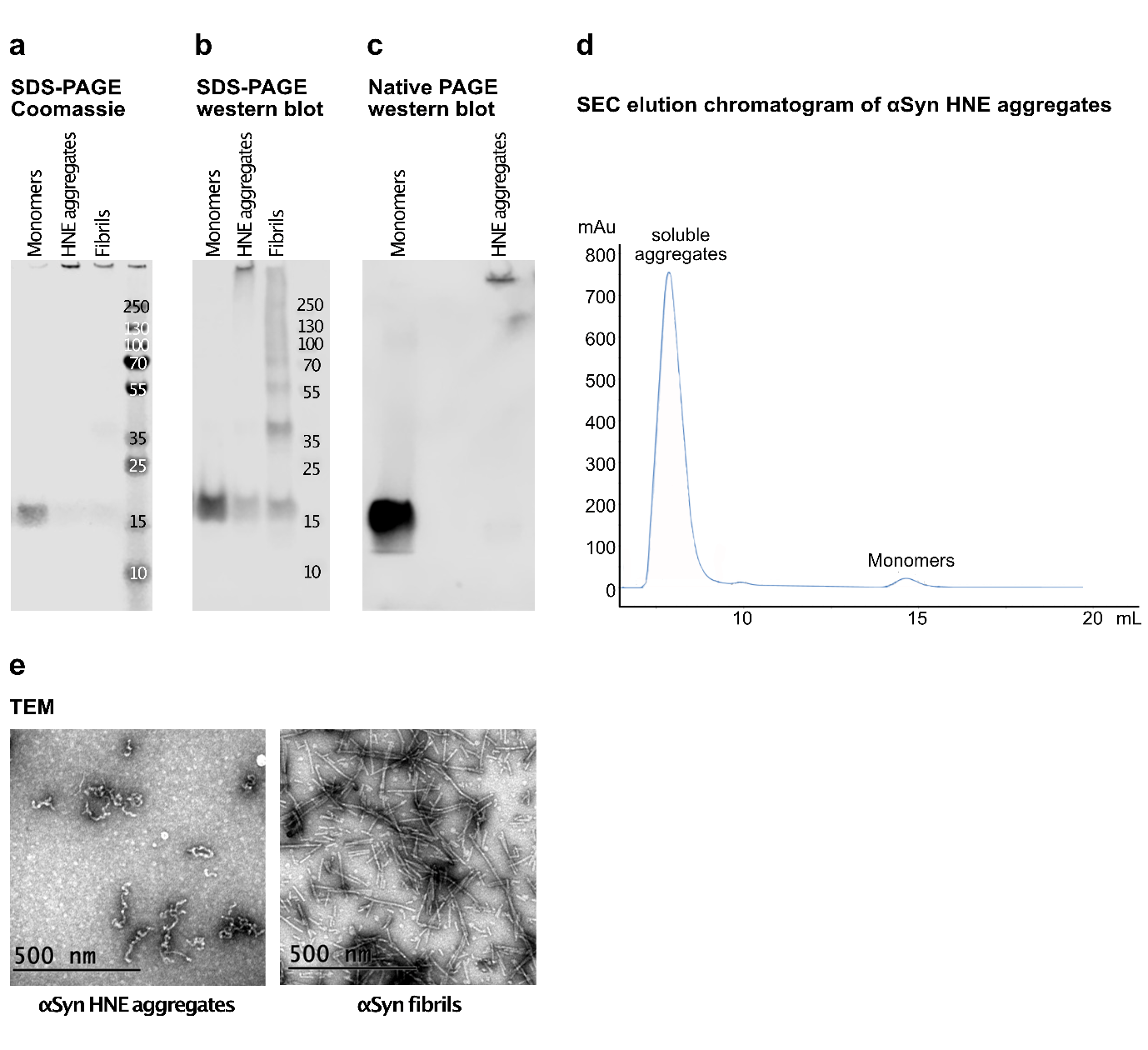
Supplementary Figure S4. Characterization of αSyn HNE aggregates and αSyn fibril preparations.** (a) SDS-PAGE with Coomassie staining with a band at ~15 kDa in the lane with αSyn monomers, bands at ~15 kDa and >250 kDa in the lane with HNE aggregates, and bands at ~15 kDa, ~ 40 kDa and >250 kDa in the lane with fibrils. 1 µg protein/lane. (b) Western blot of SDS PAGE with a band at ~15 kDa in the lane with αSyn monomers, bands at ~15 kDa, ~ 40 kDa and >250 kDa in the lane with αSyn HNE aggregates, and a ladder of bands between 15 kDa and >250 kDa in the lane with the αSyn fibril preparation. 0,25 µg protein/lane. Detection with Syn1 as primary antibody which binds all types of αSyn species. (c) Western blot of native PAGE of αSyn HNE with a band in the high molecular weight (HMW) range. 0,5 µg protein/lane. Syn1 was used as detection antibody. The complete gels and blots can be seen in Supplementary Fig. S5. (d) SEC elution chromatogram of αSyn HNE aggregates in PBS separated on a Superdex 200 Increase 10/300 GL column. The elution peaks measured by the absorbance at 280 nm indicate the presence of HMW species (calculated MW >700 kDa) and monomers (calculated MW 90 kDa). (e) TEM with negative staining shows αSyn HNE aggregates as elongated curly structures and fibrils as straight elongated structures.

**
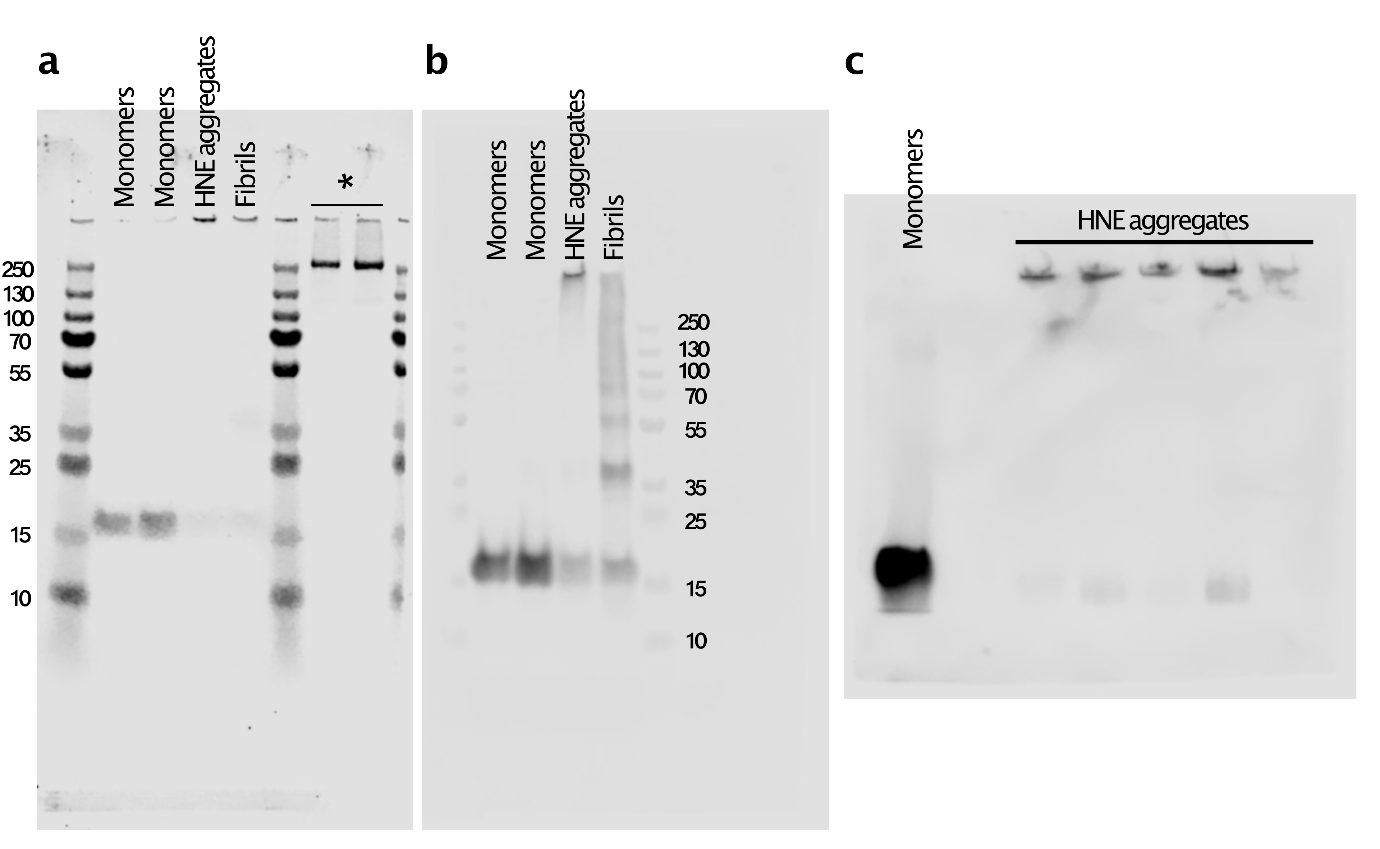
Supplementary Figure S5. Complete images of PAGEs and blots presented in Supplementary Figure S4 a-c.** (a) SDS-PAGE with Coomassie staining with αSyn monomers, αSyn HNE aggregates and αSyn fibrils. 1 µg protein/lane. Lanes marked with * are irrelevant for the project. (b) Western blot of SDS-PAGE with αSyn monomers, αSyn HNE aggregates and αSyn fibril preparation. 0,25 µg protein/lane. Detection with Syn1 as primary antibody which binds all types of αSyn species. (c) Western blot of native PAGE of αSyn monomers and αSyn HNE aggregates. 0,5 µg protein/lane. Syn1 was used as detection antibody.

**
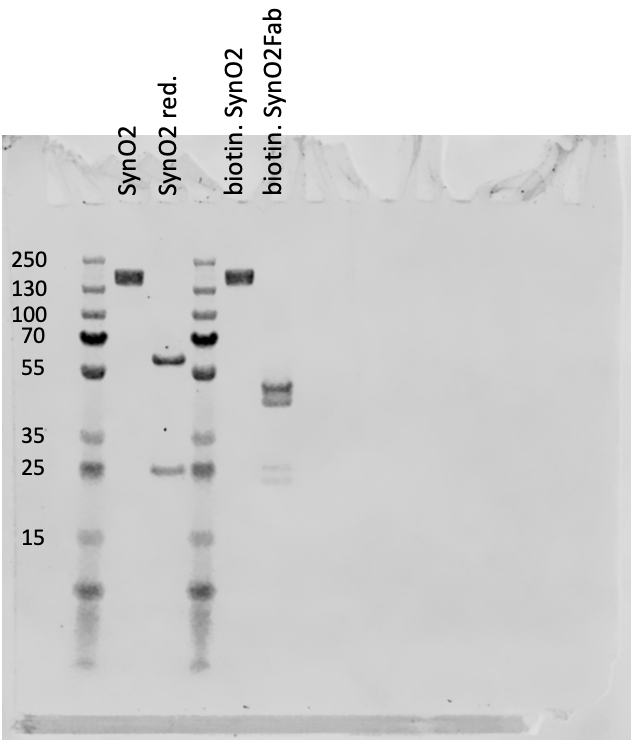
**

**Supplementary Figure S6. Complete image of Coomassie-stained SDS-PAGE presented in Figure 3 a.** Bands at 150 kDa for SynO2 and at 50 kDa for the Fab fragment SynO2Fab. A band at 25 kDa likely represents separated light and heavy Fab chains, likely caused by the reduction by cysteines in the Fab digestion buffer. 1 µg protein/lane. Lanes marked with * are irrelevant for the project.

**
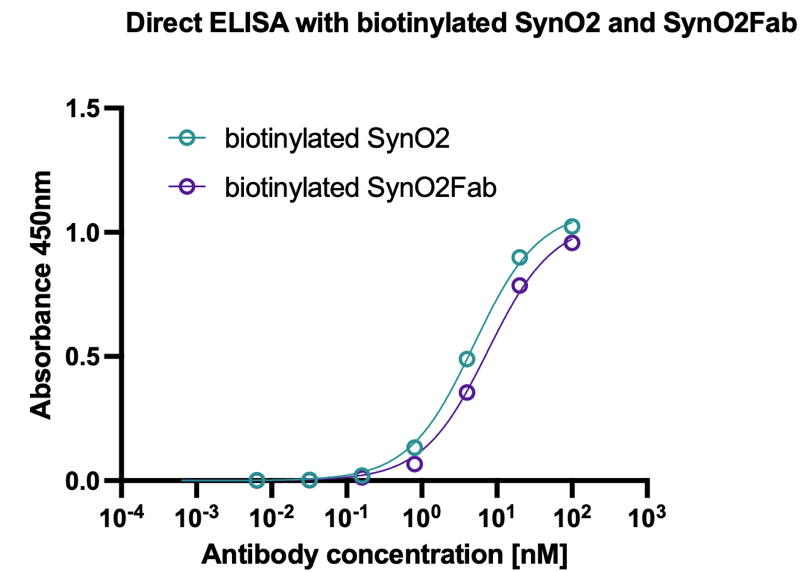
**

**Supplementary Figure S7. Direct ELISA comparing the degree of biotinylation of SynO2 and SynO2Fab.** A dilution series of biotinylated SynO2 or SynO2Fab was applied as coating and the biotinylation was detected by Streptavidin-HRP. The Fab fragment appears to have a slightly lower degree of biotinylation but the difference is far below the difference between the binding signals of biotinylated SynO2 and SynO2Fab to αSyn aggregates shown in Figure 3.

**Determination of the average degree of biotinylation using liquid chromatography mass spectrometry (LC-MS)**

To further verify that the difference in affinity between SynO2Fab and SynO2 shown with ELISA (Fig. 3c) is not due to a difference in biotinylation, the average biotinylation of the two protein complexes was determined by LC-MS. Since biotinylated, intact SynO2 antibody, containing N-linked glycosylation, was likely to be too heterogeneous to resolve and deconvolute, both intact and reduced SynO2 and SynO2Fab were analyzed. The reduction of intact SynO2 and SynO2Fab were done by incubating the protein in 5 mM dithiothreitol (DTT) at room temperature for 1 h. Samples of both reduced monomers and intact complexes were analyzed using an Acquity UPLC coupled to a SYNAPT XS (Waters). For each sample, 500 ng were injected onto a BioResolve RP mAb LC column (450Å, 2.7um, 2.1x100 mm, Waters). The proteins were separated using a gradient with the following profile: 0.0-1.0 min, 5% B; 1.1 min, 20% B; 6.0-6.5, 100% B; 6.51-10 min, 5%. The two mobile phases were: A) 0.01 % formic acid (FA) in water, and B) 0.01 % FA in acetonitrile (ACN). The column temperature was set to 60°C and the flow rate 300 nl/min. The mass spectrometer was operated in positive, resolution, MS mode, in the range of 700-3,500 m/z. The capillary voltage was set to 3.0 kV, the source temperature 120°C and the MS scan time were set to 0.5 s. Prior use, the instrument was calibrated using sodium iodide. The neutral, average masses were determined by deconvoluting the raw spectra using the MagTran software (v1.02) by Zhang and Marshall.^2^ Supplementary Fig. 8 illustrates the procedure with the SynO2Fab light chain by first separating the proteins using LC (Suppl. Fig. S8 A), combing the scans in each LC peak into raw spectra (Suppl. Fig. S8 B), followed by deconvolution (Suppl. Fig. S8 C). All proteins were analyzed in the same manner as illustrated for the SynO2Fab light chain. The different complexes and subunits were identified by matching measured and predicted masses, where the masses of the modifications were taken from www.unimod.org (Supp. Table S1-3). The relative amounts were determined from the intensities in the deconvoluted spectra. The distribution of biotinylation of intact SynO2 and SynO2Fab were calculated either using the data from the heavy chain and light chain from reduced samples, by combing all possible combinations of the biotinylated subunits, or calculated from the deconvoluted spectrum directly (intact SynO2Fab only).


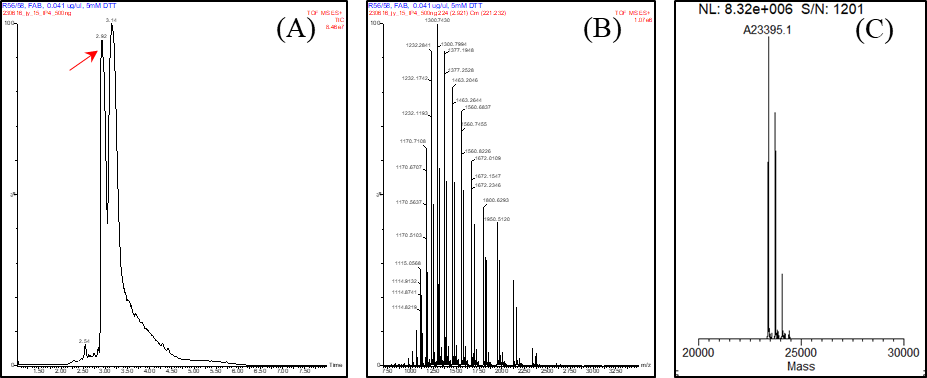


**Supplementary Figure S8. Determination of the neutral, average mass using LC-MS.** Reduced, biotinylated Fab was measured by LC-MS. The left figure (A) shows the LC trace (total ion chromatogram, with two major peaks). The spectra of the left peak (indicated by a red arrow), containing the light chain, were combined into the spectrum in (B). The spectrum contains four major charge state envelopes, formed by varying number of ionizing protons from the ionization, which can be deconvoluted into neutral average masses in (C).

As expected, it was not possible to deconvolute the intact, biotinylated SynO2 (data not shown). In contrast, intact SynO2Fab, reduced SynO2 and reduced SynO2Fab, all generated high-quality spectra (Suppl. Fig. 9 A-E). Since the reduction was done without denaturation, the interchain disulfide bonds were reduced, while the intrachain disulfide bonds stayed intact. Both SynO2 and SynO2Fab show distributions of biotinylation, and the distributions were used to calculate the average number of biotin moieties per SynO2 and SynO2Fab (Suppl. Fig. 9 F). Since the average number of biotin moieties for SynO2Fab calculated from the reduced subunits, is the same as for the intact complex (2.8), the average number of biotin moieties for SynO2 is likely to be similar to the one for the reduced complex (i.e. 3.2) (Suppl. Fig. 9 F). Hence, the data indicate that the degree of biotinylation of SynO2 (3.2) and SynO2Fab (2.8) is very similar and would likely not affect the observed affinities in ELISA (Fig. 3).

Supplementary Table S1 A: Mass measurement of reduced SynO2 light chain. Predicted mass (neutral, average; column 6) was calculated from the sequence (column 1) and modifications (columns 2-5) and compared with the measured mass (column 7; Suppl. Fig 9 A). The relative intensity (column 12) of each form was calculated from the deconvoluted spectra.

| **Predicted** | | | | | | **Measured** |  |  | **Biotin** | | |
| --- | --- | --- | --- | --- | --- | --- | --- | --- | --- | --- | --- |
| **Sequence (LC)** | **2xSS** | **pyroGlu** | **Biotin (#)** | **Biotin**  **(mass)** | **Sum** | **Mass** | **Deviation** | **Intensity** | **#** | **Int** | **%** |
|  |  |  |  |  | **(Da)** | **(Da)** | **(Da)** |  |  |  |  |
| 23414.7 | -4.0 | -17.0 | 0 | 0 | 23393.7 | 23393.4 | -0.3 | 7.32E+06 | 0 | 7.32E+06 | 77.3% |
| 23414.7 | -4.0 | -17.0 | 1 | 339.5 | 23733.2 | 23732.7 | -0.5 | 2.15E+06 | 1 | 2.15E+06 | 22.7% |

Supplementary Table S1 B: Mass measurement of reduced SynO2 (heavy chain). Predicted mass (neutral, average; column 8) was calculated from the sequence (column 1) and modifications (column 2-7) and compared with the measured mass (column 9; Suppl. Fig 9 B). The relative intensity (column 14) of each form was calculated from the deconvoluted spectra.

| **Predicted** | | | | | | | | | **Measured** |  |  | **Biotin** | | |
| --- | --- | --- | --- | --- | --- | --- | --- | --- | --- | --- | --- | --- | --- | --- |
| **Sequence (HC)** | **4xSS** | **C-term K** | **Glycosylation** | | **Biotin (#)** | | **Biotin**  **(mass)** | **Sum** | **Mass** | **Deviation** | **Intensity** | **#** | **Int** | **%** |
|  |  |  | **G0F** | **G1F** | |  |  | **(Da)** | **(Da)** | **(Da)** |  |  |  |  |
| 49876.4 | -8.0 | -128.2 | 1445.3 |  | | 0 | 0 | 51185.5 | 51186 | 0.5 | 5.57E+05 | 0 |  |  |
| 49876.4 | -8.0 | -128.2 |  | 1607.5 | | 0 | 0 | 51347.7 | 51349 | 1.3 | 6.20E+05 | 0 | 1.18E+06 | 17.3% |
| 49876.4 | -8.0 | -128.2 | 1445.3 |  | | 1 | 339.5 | 51525.0 | 51526 | 1.0 | 1.25E+06 | 1 |  |  |
| 49876.4 | -8.0 | -128.2 |  | 1607.5 | | 1 | 339.5 | 51687.2 | 51688 | 0.8 | 1.35E+06 | 1 | 2.60E+06 | 38.3% |
| 49876.4 | -8.0 | -128.2 | 1445.3 |  | | 2 | 679 | 51864.5 | 51865 | 0.5 | 1.16E+06 | 2 |  |  |
| 49876.4 | -8.0 | -128.2 |  | 1607.5 | | 2 | 679 | 52026.7 | 52028 | 1.3 | 1.12E+06 | 2 | 2.28E+06 | 33.6% |
| 49876.4 | -8.0 | -128.2 | 1445.3 |  | | 3 | 1018.5 | 52204.0 | 52203 | -1.0 | 4.55E+05 | 3 |  |  |
| 49876.4 | -8.0 | -128.2 |  | 1607.5 | | 3 | 1018.5 | 52366.2 | 52367 | 0.8 | 2.81E+05 | 3 | 7.36E+05 | 10.8% |

Supplementary Table S2 A: Mass measurement of reduced SynO2Fab light chain. Predicted mass (neutral, average; column 6) was calculated from the sequence (column 1) and modifications (column 2-5) and compared with the measured mass (column 7; Suppl. Fig 9 C). The relative intensity (column 12) of each form was calculated from the deconvoluted spectra.

| **Predicted** | | | | | | **Measured** |  |  | **Biotin** | | |
| --- | --- | --- | --- | --- | --- | --- | --- | --- | --- | --- | --- |
| **Sequence (LC)** | **2xSS** | **pyroGlu** | **Biotin (#)** | **Biotin**  **(mass)** | **Sum** | **Mass** | **Deviation** | **Intensity** | **#** | **Int** | **%** |
|  |  |  |  |  | **(Da)** | **(Da)** | **(Da)** |  |  |  |  |
| 23414.7 | -4.0 | -17.0 | 0 | 0 | 23393.7 | 23395.1 | 1.4 | 8.32E+06 | 0 | 8.32E+06 | 50.2% |
| 23414.7 | -4.0 | -17.0 | 1 | 339.5 | 23733.2 | 23733.2 | 0 | 6.24E+06 | 1 | 6.24E+06 | 37.7% |
| 23414.7 | -4.0 | -17.0 | 2 | 679 | 24072.7 | 24073.4 | 0.7 | 1.78E+06 | 2 | 1.78E+06 | 10.7% |
| 23414.7 | -4.0 | -17.0 | 3 | 1018.5 | 24412.2 | 24412.7 | 0.5 | 2.28E+05 | 3 | 2.28E+05 | 1.4% |

Supplementary Table S2 B: Mass measurement of reduced SynO2Fab heavy chain. Predicted mass (neutral, average; column 5) was calculated from the sequence (column 1) and modifications (column 2-4) and compared with the measured mass (column 6; Suppl. Fig 9 D). The relative intensity (column 11) of each form was calculated from the deconvoluted spectra.

| **Predicted** | | | | | **Measured** |  |  | **Biotin** | | |
| --- | --- | --- | --- | --- | --- | --- | --- | --- | --- | --- |
| **Sequence (HC)** | **2xSS** | **Biotin (#)** | **Biotin**  **(mass)** | **Sum** | **Mass** | **Deviation** | **Intensity** | **#** | **Int** | **%** |
|  |  |  |  | **(Da)** | **(Da)** | **(Da)** |  |  |  |  |
| 24279.1 | -4.0 | 0 | 0 | 24275.1 | 24276.1 | 1 | 7.97E+05 | 0 | 7.97E+05 | 5.9% |
| 24279.1 | -4.0 | 1 | 339.5 | 24614.6 | 24616.3 | 1.7 | 3.23E+06 | 1 | 3.23E+06 | 23.8% |
| 24279.1 | -4.0 | 2 | 679 | 24954.1 | 24954.6 | 0.5 | 4.41E+06 | 2 | 4.41E+06 | 32.5% |
| 24279.1 | -4.0 | 3 | 1018.5 | 25293.6 | 25294 | 0.4 | 3.26E+06 | 3 | 3.26E+06 | 24.0% |
| 24279.1 | -4.0 | 4 | 1358 | 25633.1 | 25634 | 0.9 | 1.54E+06 | 4 | 1.54E+06 | 11.3% |
| 24279.1 | -4.0 | 5 | 1697.5 | 25972.6 | 25973 | 0.4 | 3.45E+05 | 5 | 3.45E+05 | 2.5% |

Supplementary Table S3: Mass measurement of intact SynO2Fab. Predicted mass (neutral, average; column 7) was calculated from the sequence (column 1-2) and modifications (column 3-6) and compared with the measured mass (column 8; Suppl. Fig 9 E). The relative intensity (column 13) of each form was calculated from the deconvoluted spectra.

| **Predicted** | | | | | | | **Measured** |  |  | **Biotin** | | |
| --- | --- | --- | --- | --- | --- | --- | --- | --- | --- | --- | --- | --- |
| **Sequence** | | **4xSS** | **pyroGlu** | **Biotin (#)** | **Biotin (mass)** | **Sum** | **Mass** | **Deviation** | **Intensity** | **#** | **Int** | **%** |
| **(LC)** | **(HC:**  **1-224)** |  |  |  |  | **(Da)** | **(Da)** | **(Da)** |  |  |  |  |
| 23414.7 | 24279.1 | -8.0 | -17.0 | 0 | 0.0 | 47668.8 | n.d. | n.a. | n.d. | 0 | 0.00E+00 | 0.0% |
| 23414.7 | 24279.1 | -8.0 | -17.0 | 1 | 339.5 | 48008.3 | 48009 | 0.7 | 1.96E+06 | 1 | 1.96E+06 | 17.9% |
| 23414.7 | 24279.1 | -8.0 | -17.0 | 2 | 679.0 | 48347.8 | 48348 | 0.2 | 2.95E+06 | 2 | 2.95E+06 | 26.9% |
| 23414.7 | 24279.1 | -8.0 | -17.0 | 3 | 1018.5 | 48687.3 | 48688 | 0.7 | 2.83E+06 | 3 | 2.83E+06 | 25.8% |
| 23414.7 | 24279.1 | -8.0 | -17.0 | 4 | 1358.0 | 49026.8 | 49027 | 0.2 | 2.08E+06 | 4 | 2.08E+06 | 19.0% |
| 23414.7 | 24279.1 | -8.0 | -17.0 | 5 | 1697.5 | 49366.3 | 49367 | 0.7 | 9.46E+05 | 5 | 9.46E+05 | 8.6% |
| 23414.7 | 24279.1 | -8.0 | -17.0 | 6 | 2037.0 | 49705.8 | 49705 | -0.8 | 2.06E+05 | 6 | 2.06E+05 | 1.9% |


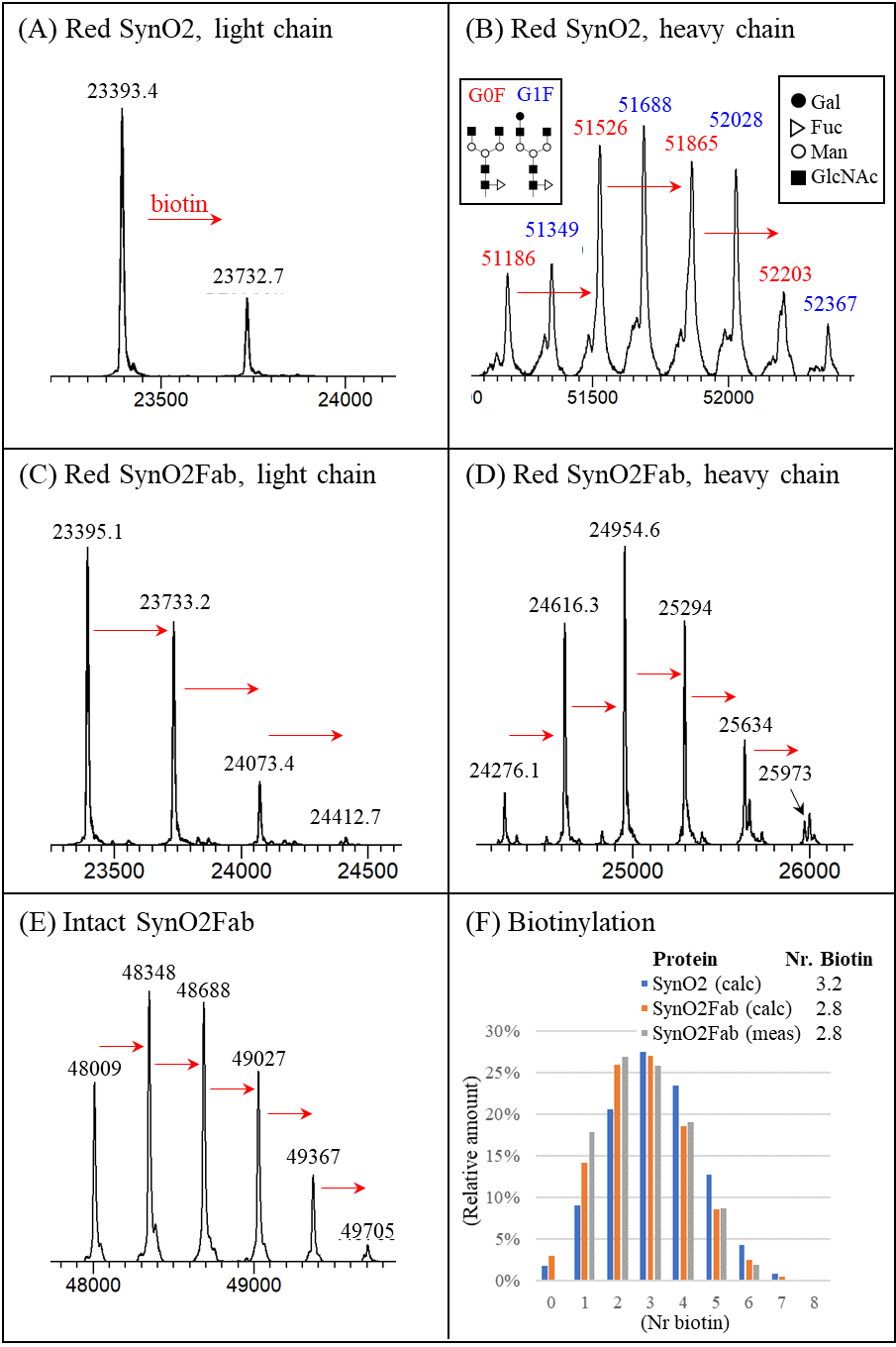


**Supplementary Figure S9. Determination of the neutral, average mass using LC-MS.** The figure shows the deconvoluted masses of reduced SynO2 (A-B), reduced SynO2Fab (C-D) and intact SynO2Fab (E) measured by LC-MS. The intensities are based on the charge states envelopes in the raw spectra. The intact masses of all protein forms with matched masses are indicated in the figure and listed in Supplementary Tables 1-3. Red arrows indicate peaks shifted by the mass of a biotin moiety (339.5 Da). The intact, reduced heavy chain from SynO2 (B), contains N-linked glycosylation, where the G0F (red) and G1F (blue) constitute the major forms. The schematic carbohydrate structures are shown in the upper left inset in figure (B), with the key to the right. The intensities of the deconvoluted protein forms were used to calculate the distribution of biotin moieties per SynO2 and SynO2Fab, and compared to the measured number of biotin moieties (F). Blue corresponds to calculated values for SynO2, red for calculated SynO2Fab, and grey for measured SynO2Fab. The y-axis shows the relative amount in percent and the x-axis the number of biotin moieties. The data was used to calculate the average number of biotin moieties per SynO2 and SynO2Fab (F, inset).

**
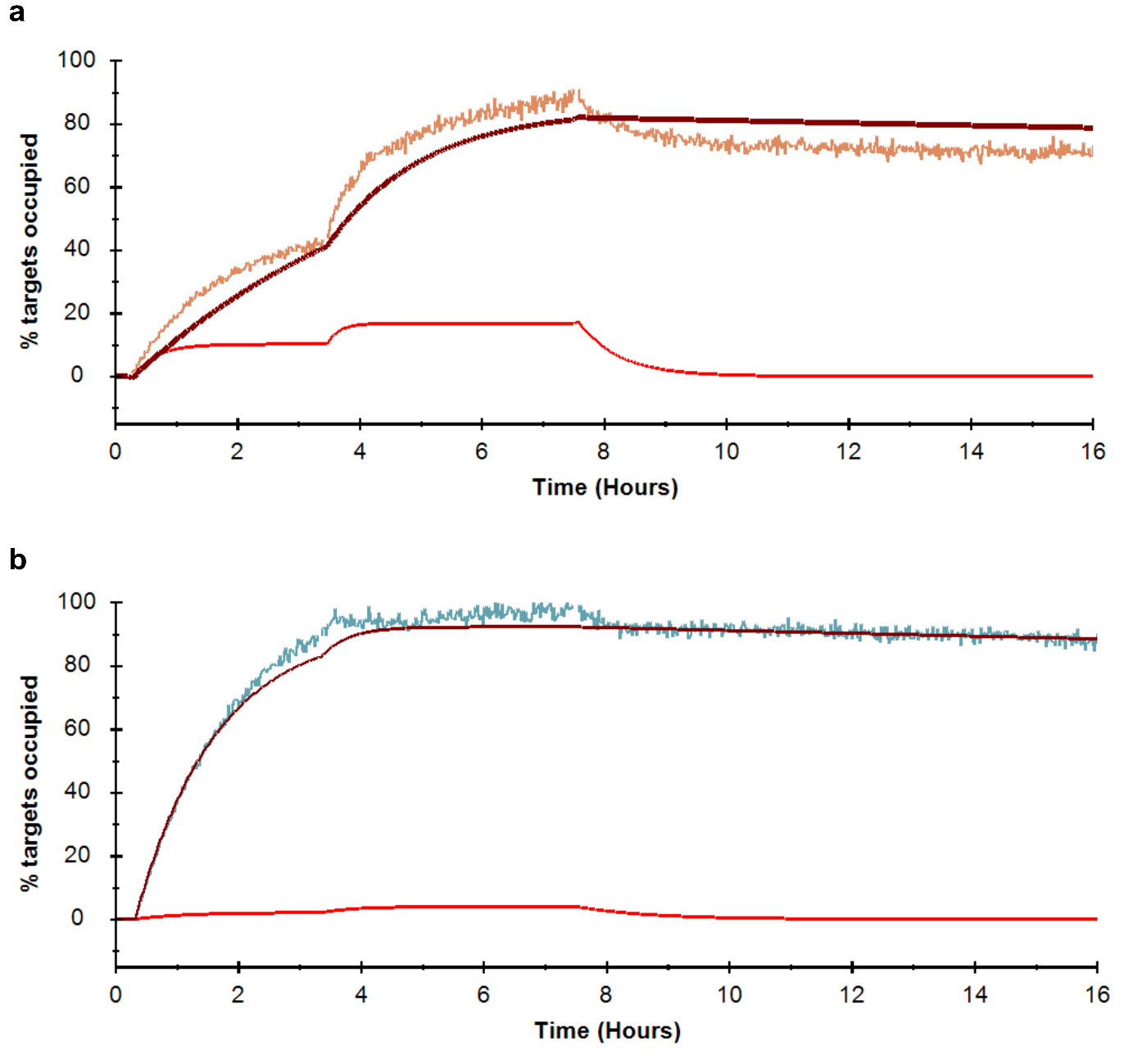
**

**Supplementary Figure S10. Interaction curves of (a) SynO2 and (b) TetraSynO2 with αSyn HNE aggregates recorded by LigandTracer with one-to-two fitting curves split into the weak (light red) and the strong (dark red) interaction component, respectively.** 100 nM coating with αSyn HNE aggregates. Two consecutive association phases (3 hours and 4 hours respectively) with 1 nM and 3 nM of the respective ^125^I-labelled antibody. Recorded interaction curves were evaluated in TraceDrawer using a. Signal intensities of each curve were scaled to Bmax, the estimated signal intensity at saturation, with 100% representing target saturation.

Supplementary references

1. Almandoz-Gil L, Welander H, Ihse E, Khoonsari PE, Musunuri S, Lendel C, Sigvardson J, Karlsson M, Ingelsson M, Kultima K, et al. Low Molar Excess of 4-Oxo-2-Nonenal and 4-Hydroxy-2-Nonenal Promote Oligomerization of Alpha-Synuclein through Different Pathways. *Free Radic. Biol. Med.* 2017;110:421–431, doi:10.1016/j.freeradbiomed.2017.07.004.

2. Zhang Z, Marshall AG. A Universal Algorithm for Fast and Automated Charge State Deconvolution of Electrospray Mass-to-Charge Ratio Spectra. *J. Am. Soc. Mass Spectrom.* 1998;9:225–233, doi:10.1016/S1044-0305(97)00284-5.
